# Consuming microplastics? Investigation of commercial salts as a source of microplastics (MPs) in diet

**DOI:** 10.1007/s11356-022-22101-0

**Published:** 2022-07-30

**Authors:** Aswin Kuttykattil, Subash Raju, Kanth Swaroop Vanka, Geetika Bhagwat, Maddison Carbery, Salom Gnana Thanga Vincent, Sudhakaran Raja, Thava Palanisami

**Affiliations:** 1 Environmental Plastics Innovation Cluster (EPIC), Global Innovative Centre for Advanced Nanomaterial (GICAN), The University of Newcastle, Callaghan, Newcastle, NSW 2308 Australia; 2 School of Biomedical Sciences and Pharmacy, The University of Newcastle/Priority Research Centre for Healthy Lungs, Hunter Medical Research Institute, The University of Newcastle, Newcastle, NSW Australia; 3 Department of Environmental Sciences, University of Kerala, Kerala, India; 4 Aquaculture Biotechnology Laboratory, School of Bio-Sciences and Technology, Vellore Institute of Technology, Vellore, India

**Keywords:** Microplastics, Fibres, Commercial salt, Salt consumption, Human health

## Abstract

The omnipresence of microplastics (MPs) in marine and terrestrial environments as a pollutant of concern is well established and widely discussed in the literature. However, studies on MP contamination in commercial food sources like salts from the terrestrial environment are scarce. Thus, this is the first study to investigate various varieties of Australian commercial salts (both terrestrial and marine salts) as a source of MPs in the human diet, and the first to detect MPs in black salt. Using Nile red dye, the MPs were detected and counted under light microscopy, further characterised using attenuated total reflectance Fourier transformed infrared spectroscopy (ATR-FTIR) and scanning electron microscopy and energy-dispersive X-ray spectroscopy (SEM–EDS). Of all the 90 suspected particles, 78.8% were identified as MPs with a size ranging between 23.2 µm and 3.9 mm. The fibres and fragments constituted 75.78% and 24.22% respectively. Among the tested samples, Himalayan pink salt (coarse) from terrestrial sources was found to have the highest MP load, i.e. 174.04 ± 25.05 (SD) particle/kg, followed by black salt at 157.41 ± 23.13 particle/kg. The average concentration of detected MPs in Australian commercial salts is 85.19 ± 63.04 (SD) per kg. Polyamide (33.8%) and polyurethane (30.98%) were the dominant MP types. Considering the maximum recommended (World Health Organization) salt uptake by adults daily at 5 g, we interpret that an average person living in Australia may be ingesting approximately 155.47 MPs/year from salt uptake. Overall, MP contamination was higher in terrestrial salts (such as black and Himalayan salt) than the marine salt. In conclusion, we highlight those commercial salts used in our daily lives serve as sources of MPs in the diet, with unknown effects on human health.

**Supplementary Information:**

The online version contains supplementary material available at 10.1007/s11356-022-22101-0.

## Introduction

MPs (< 5 mm) are omnipresent and are well-established pollutants contaminating both terrestrial and aquatic habitats. Unlike the macroplastic debris, the MPs spread across the landfills or freshwater or marine or estuaries cannot be easily detected by the naked eye but pose a serious threat to environmental and human health (Browne et al. 2010; Claessens et al. 2011; Cole et al. 2011; Law et al. 2010; Prata 2018; Thompson et al. 2004; Zhao et al. 2014). Based on the origin, the MP’s pollutants in nature can be classified as primary and secondary MPs. Primary MPs consist of industrially manufactured particles that are intentionally produced at the microscale to suit human needs, e.g. microbeads used in toothpaste, beauty care products. In contrast, secondary MPs are the products of natural/artificial weathering of macroplastic debris (GESAMP 2015; Koelmans et al. 2014).

Human activities like fishing, nautical, aquaculture, and other maritime sources such as shipwrecks have exponentially contributed to marine plastic litter (MPL) (Lusher et al. 2017). The distinct properties like buoyancy, durability, and low sedimentation rate enable MPL to remain persistent in the marine environment and facilitate their transport to considerable distances aided by oceanic currents (Cole et al. 2016). Wastewater treatment plants (WTP) are other sources of MPL; studies show that an estimated 10% of the plastics produced annually escape into oceans through wastewater treatment plants (Raju et al. 2018; Zhao et al. 2017). A global oceanic survey (in South Pacific, North Pacific, South Atlantic, Indian Ocean, and waters around Australia) reports the abundance of marine plastic debris from 7000–35,000 metric tonnes (MT) to 268,940 MT, thus demonstrating a major global environmental issue (Iniguez et al. 2018; Zhao et al. 2017).

Increased MPL or MPs in seas/oceans leads to contamination of seafood reserves with MPs, which reaches the human gut upon ingestion (Yang et al. 2015). Accumulation of MPs in sea salts (Zhang et al. 2020), fish (Thiele et al. 2021), mussels (Li et al. 2018), shrimps (Gurjar et al. 2021), etc. will have a serious implication for humans worldwide due to food safety concern (Curren et al. 2020; Lusher et al. 2017). In addition to sea salt, a few other edible salts are derived from aquatic (lake, river, and groundwater) and terrestrial (rock salt) regions. Plastic contamination of landfills, freshwater sources, or handling activities in industries will likely introduce the MPs into the products. MP contamination from the atmospheric deposition of the airborne MPs (Allen et al. 2019), mechanical breakage of the plastic material during the manufacturing process (Cooper 2007), and airborne microfibre from the clothes of the industrial workers (Prata 2018) are other potential sources of contamination in salts. The MPs may also end in salt from the industrial equipment or material (Dris et al. 2016), the release of wear particles from conveyor belts (Andrejiova et al. 2016; Bindzár 2002), furniture inside the industries, or from the tear and wear of synthetic tyres from vehicles (Prata 2018). Automobile seats are the reservoir of textile fibres (Roux and Margot 1997), and these textile fibres can travel through the air during wind (Horton et al. 2017).

Salt types are extracted by different methods; for instance, evaporation is preferred for sea salt and lake salt, rock salt is mined from mineral rocks (halite), whereas river salt and well salt are extracted from wells in non-coastal areas (Iniguez et al. 2018). Rock salts are known for their traditional use as a spice, flavour enhancer in foods, and natural supplements. The refined version is table salt (or iodised salt), consisting of 97–99% sodium chloride (NaCl). Himalayan pink salt or Himalayan salt is mined in Salt Mines, Pakistan. It comprises 95–98% NaCl, 2–4% polyhalite (potassium, calcium, magnesium, sulphur, oxygen, hydrogen), 0.01% fluoride, 0.01% iodine, and a small number of various trace minerals. Himalayan salts are exported to several countries, including Australia, and used similarly as table salts (Sarker et al. 2016). Black salt is commonly composed of NaCl with trace impurities of sodium sulphate, sodium bisulphate, sodium sulphide, iron sulphide, hydrogen sulphide, and sodium bisulphite. Sea salts are the purest form of rock salt formed from the evaporation of salty water from estuaries, enclosed bays, and inland marginal seas in semi-arid areas (Sarker et al. 2016).

Consumption of commercial salts denotes a risk of MP introduction in the gastrointestinal tracts (GIT), which may have potential complications to human health (Wibowo et al. 2021; Yan et al. 2022). Ingested MPs < 150 µm in size generate toxicity in the secondary organs and tissues (Yuan et al. 2022), whereas > 150 µm may induce damage in the gut (EFSA 2016). However, these local effects are understudied. An oral exposure study in mice that ingested polystyrene (PS) MPs (1000 μg/L) for 5 weeks demonstrated decreased body and liver weights (Lu et al. 2018). Further, the study also emphasised that MPs may reduce the gut mucus secretion and disturb the gut microbiota internally. In addition, MPs are bioavailable to marine organisms at all levels of the food chain (Barboza et al. 2018; Carbery et al. 2018; Nelms et al. 2018; Walkinshaw et al. 2020). Due to their composition and significantly wider surface area, MPs are prone to attracting waterborne organic contaminants, microbes, and leaching of harmful plasticisers or additives (Bhagwat et al. 2021; Foulon et al. 2016; Joo et al. 2021; Schrank et al. 2019; Wang et al. 2015). Ingestion of microplastics may thereby introduce toxins to the food chain’s base, where they can bioaccumulate (Teuten et al. 2009). Contaminants (Bakir et al. 2016; Koelmans et al. 2016) and microbes (Foulon et al. 2016) adhered to MPs may have negative consequences for humans who consume contaminated foods. As a result, the presence of MPs in human food is a concern for food safety (Diogo Peixot et al. 2019).

World Health Organization (WHO) recommends 5 g/day as maximum salt consumption in adults (WHO 2012). However, this value exceeds in most countries, and global salt intake may be estimated at an average of 10 g/day (Mozaffarian et al. 2014). A systematic review and meta-analysis data revealed that Australian men have average salt consumption per day of 10.1 g/day, and women consume 7.34 g/day (Land et al. 2018).

Currently, the knowledge of the presence of MP contamination in salts from terrestrial sources is understudied. Thus, to investigate the commercial salts as a source of microplastics, we have selected seven commercial salt types (both marine and terrestrial salts) from the shelves of supermarkets in Australia that are commonly used in everyday cooking and estimated the particle number, types, and nature in this article. Moreover, this is the first study to investigate MP contamination in black salts, and to the best of our knowledge, no studies are comparing the presence of MP in various salts used in Australia.

## Materials and methods

### Microplastic isolation and visualisation

**Fig. 1:**
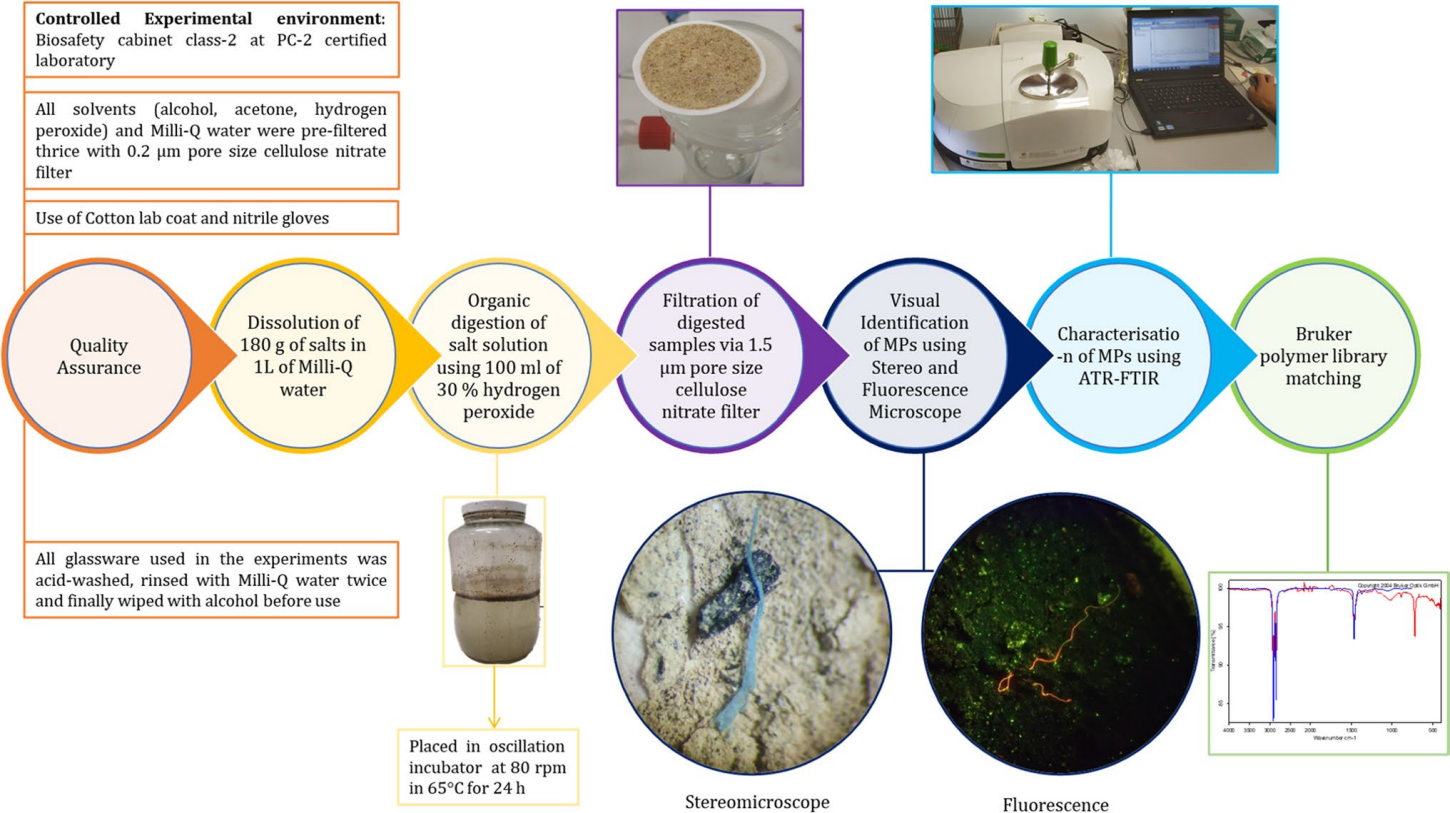
Flow diagram for microplastic extraction from salt samples

Seven different representative salt types—salt A (table salt [TS]), salt B (black salt [BS]), salt C (sea salt [SS]), salt D (iodised salt [IS]), salt E (rock salt [RS]), salt F (Himalayan pink salt (fine) [HPF]), and salt G (Himalayan pink salt (coarse) [HPC]), were collected from local supermarkets from Newcastle, Australia (Table 1). All experiments were conducted in a controlled environment within a biosafety cabinet class 2 (Model number US218D, Gelaire, Australia) in a PC-2-certified laboratory. All glassware used in the study was acid-washed and free of MPs. To facilitate a complete salt dissolution, 180 g of each salt sample was dissolved in 1 L of Milli-Q water and mixed thoroughly until the salt was completely dissolved. A protocol modified from Yang et al. (2015) was followed for MP extraction, which involved a pre-treatment of salt solution with 100 mL of 30% H_2_O_2_ to digest the organic matter. The bottles were capped and placed in an oscillation incubator at 65 °C operated at 80 rpm for 24 h and subsequently at room temperature for 48 h (Yang et al. 2015). The salt solution was immediately filtered through a Buchner funnel through a 1.5-µm pore size cellulose nitrate filter (47 mm diameter) paper using a glass vacuum filtration apparatus. The filter paper was dried inside the biosafety cabinet at room temperature, and coloured MPs were visualised under a stereomicroscope (Leica wild Heerbrugg M3B, Switzerland). For identifying transparent particles, the filter papers were stained with 10 µg/mL Nile red (9-diethylamino-5-benzo[α]phenoxazinone) prepared in acetone and dried inside the biosafety cabinet at room temperature. Excess stain in filter paper was removed by repeated washing with filtered Milli-Q water. NIGHTSEA® Illumination system with a royal blue beam (440–460 nm) and orange filters (500 nm) in stereomicroscope (Leica wild Heerbrugg M3B, Switzerland, 40 × magnification) was used to observe the stained particles. A summary of the methodology section is illustrated in Fig. 1.

**Table 1:** Background information for the salt samples used in the study

S. no	Sample ID	Type of salt	ID	Packaging material	Origin country	Uses
1	Salt A	Table salt (fine)	TS	Whole body-hard paper	Australia	Cooking, seasoning, preserving food
2	Salt B	Rock salt (black)	BS	Whole body-PE	India	Garnishing salads, a component of chat masala
3	Salt C	Sea salt (coarse)	SS	Whole body-PE	Australia	Seasoning in foods, cooking, cosmetics
4	Salt D	Iodised salt	IS	Whole body-high-density polyethylene (HDPE)	Australia	Used in cooking to avoid iodine deficiency in humans
5	Salt E	Mined (rock salt)	RS	Whole body-PE	Australia	Cooking, ice cream production, seasoning, and preserving
6	Salt F	Himalayan salt (fine)	HPF	Whole body-PE	Pakistan	Melt the ice in sidewalks and driveways after snowstorms, cooking, and salads
7	Salt G	Himalayan salt (coarse)	HPC	Whole body-high-density polyethylene (HDPE)	Pakistan	Melting ice

### Identification and characterisation of MPs

MPs were identified and characterised based on the properties such as colour, shape (fragment or fibre), and size using the stereomicroscope (Leica wild Heerbrugg M3B, Switzerland) with appropriate scale bars. Further, high-resolution images (40 ×) were captured and analysed using ImageJ software. The images were first converted to 8 bits; scales were calibrated using the filter paper diameter (47 mm) and then analysed with the measurement tool. Representative particles of suspected MPs from salt samples were investigated using a ZEISS EVO 18 scanning electron microscopy (SEM) combined with energy-dispersive X-ray spectroscopy (EDS), allowing elemental composition determination. Briefly, particles were placed and sputter-coated with gold on a 0.9 cm × 0.7 cm double-sided adhesive carbon disc (0.1 cm thickness), mounted on an aluminium stab. The images were taken at 10 kV accelerated voltage with varying magnifications.

The sample stage in ATR-FTIR (Perkin Elmer Spectrum Two) was cleaned with acetone followed by baseline correction. The particles exhibiting plastic-like nature (> 20 µm in size) on the filter paper were randomly selected using a syringe needle and placed in the sample stage having a LiTaO_3_ detector for ATR-FTIR (Perkin Elmer Spectrum Two) analysis in transmittance mode with spectra recording from 4000 to 675 cm^−1^ with 4 cm^−1^ resolutions. All the spectra obtained from the ATR-FTIR were compared with Bruker polymer library. Spectral matches with quality index scores of ~ 700 or more were considered quality matches.

The microplastic ingestion from salt consumption per year was calculated by a modified equation from (Senathirajah et al. 2021):

$${MPs}_{ISY}= \frac{\propto \times \beta \times 365}{1000}$$

where ${MPs}_{ISY}$ is the MPs ingested from salt in a year (MPs/year); $\propto$ represents the mean MP concentration (particles/kg) in salt samples; and $\beta$ indicates the daily salt consumption (g) for adults. Daily salt consumption was adapted from the WHO recommendation of 5 g daily salt intake by humans (Mozaffarian et al. 2014). Daily salt consumption (g) for Australian men and female were adapted from a systematic review and meta-analysis of data on salt consumption by Land et al. (2018).

### Quality control and statistical analysis

All the experiments were conducted in triplicates. The quality control and assessment were done based on Raju et al. (2020). In brief, extraction and processing of MPs were conducted in a controlled environment within a biosafety cabinet class 2 (Model number US218D, Gelaire, Australia) in a PC-2-certified laboratory to prevent contamination with airborne particles (Karami et al. 2017). The Milli-Q water and other solvents (acetone, alcohol, and hydrogen peroxide) for the entire experiment were pre-filtered thrice with a 0.2-µm pore size cellulose nitrate membrane filter and were stored separately in sealed glass containers. All glassware used in the experiments was acid-washed, rinsed with Milli-Q water twice, and finally wiped with alcohol before use. Cotton lab coat and nitrile gloves were worn during the entire duration of experiment. The statistical analysis was carried out using GraphPad Prism version 8. Two-way ANOVA analysis with Sidak test was selected for multiple comparisons.

A procedural blank containing only filtered Milli-Q water was tested simultaneously using the same extraction procedure as of samples (Karami et al. 2017). This control was run in triplicates for each different sample.

## Results

### Abundance, type, and size of MPs in commercial salts

**Fig. 2:**
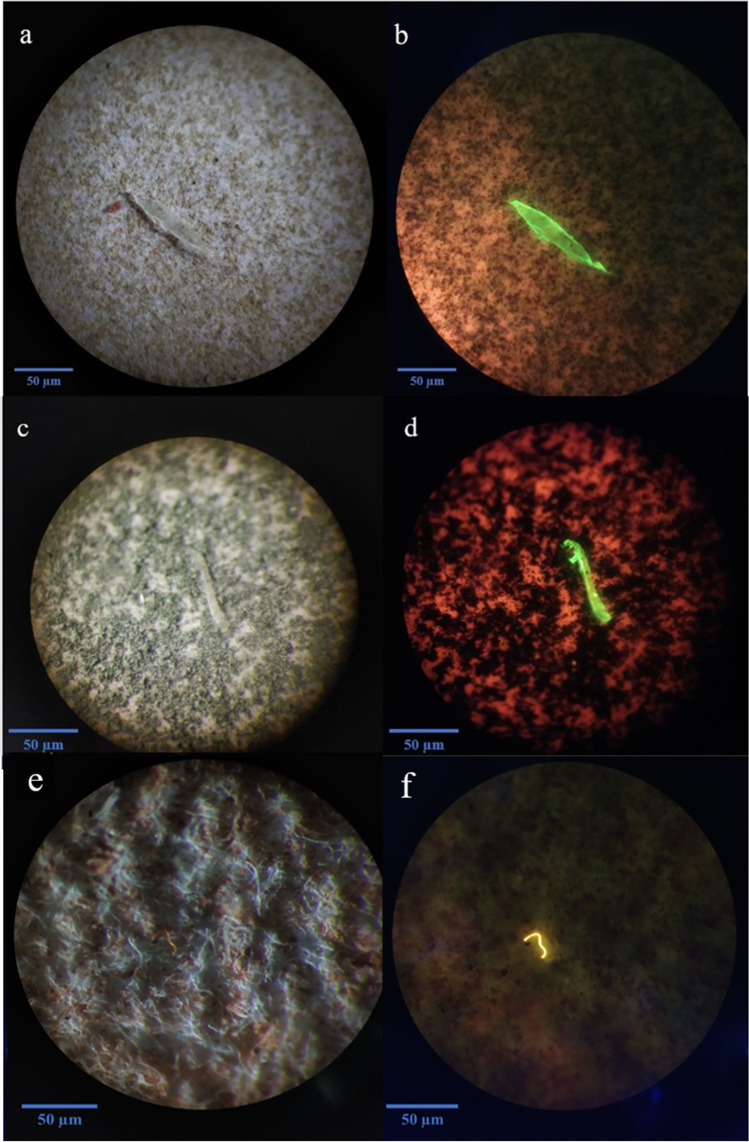
Microscopic and fluorescence images of isolated microplastics. **a** Clear fragment from Himalayan pink salt (salt G). **b** Fluorescence image of MPs in salt G. **c** Clear fragment from sea salt (salt C). **d** Fluorescent image of MPs in salt C. **e** Microscopic image of yellow fibre from black salt (salt B). **f** Fluorescent image of MPs in salt B. All the images were captured at 40 × magnification

**Fig. 3:**
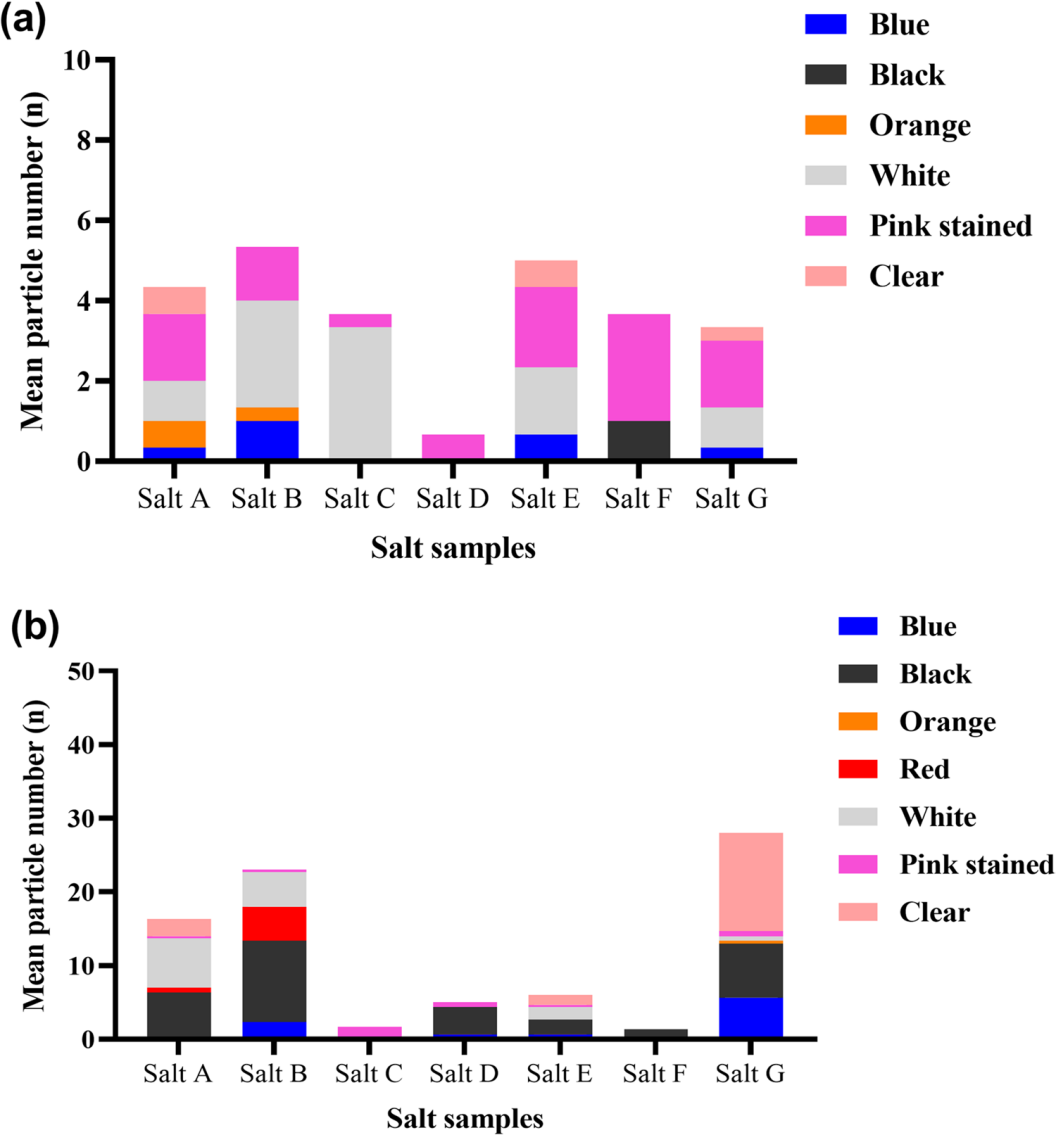
Colour profile of MP **a** fragments and **b** fibres observed from salt samples (in 180 g of salt sample)

The microscopic analysis revealed the presence of coloured, transparent, fluorescent fibres and fragments in all the salt samples (Fig. 2, Fig. S1). No particles were observed in the negative control. Few transparent and white particles absorbed the Nile red stain, which imparted a pinkish colour to these particles. All the pink and pinkish particles were categorised as “Pink stained” (supplementary table S1). Black and blue were prominent among the different coloured fibres and fragments (Fig. 3), whereas white and pink were the most common fragments. The observed MP concentration in IS, RS, SS, HPC, BS, TS, and HPF samples was 31.48 ± 16.97 (standard deviation [SD]), 61.11 ± 19.25, 29.63 ± 13.98, 174.07 ± 25.05, 157.41 ± 23.13, 114.81 ± 13.98, and 27.78 ± 14.70 particles/kg of salt, respectively. The average amount of MP contamination in 1 kg of commercial salts in Australia is 85.19 ± 63.04 (SD) particles. WHO recommends a daily 5 g intake of salts for adults; based on our finding, the average MP concentration would be 0.4259 particles/5 g of salt. For 1 year, the MP concentration in Australian commercial salt would be approximately 155.47 particles per year.

**Fig. 4:**
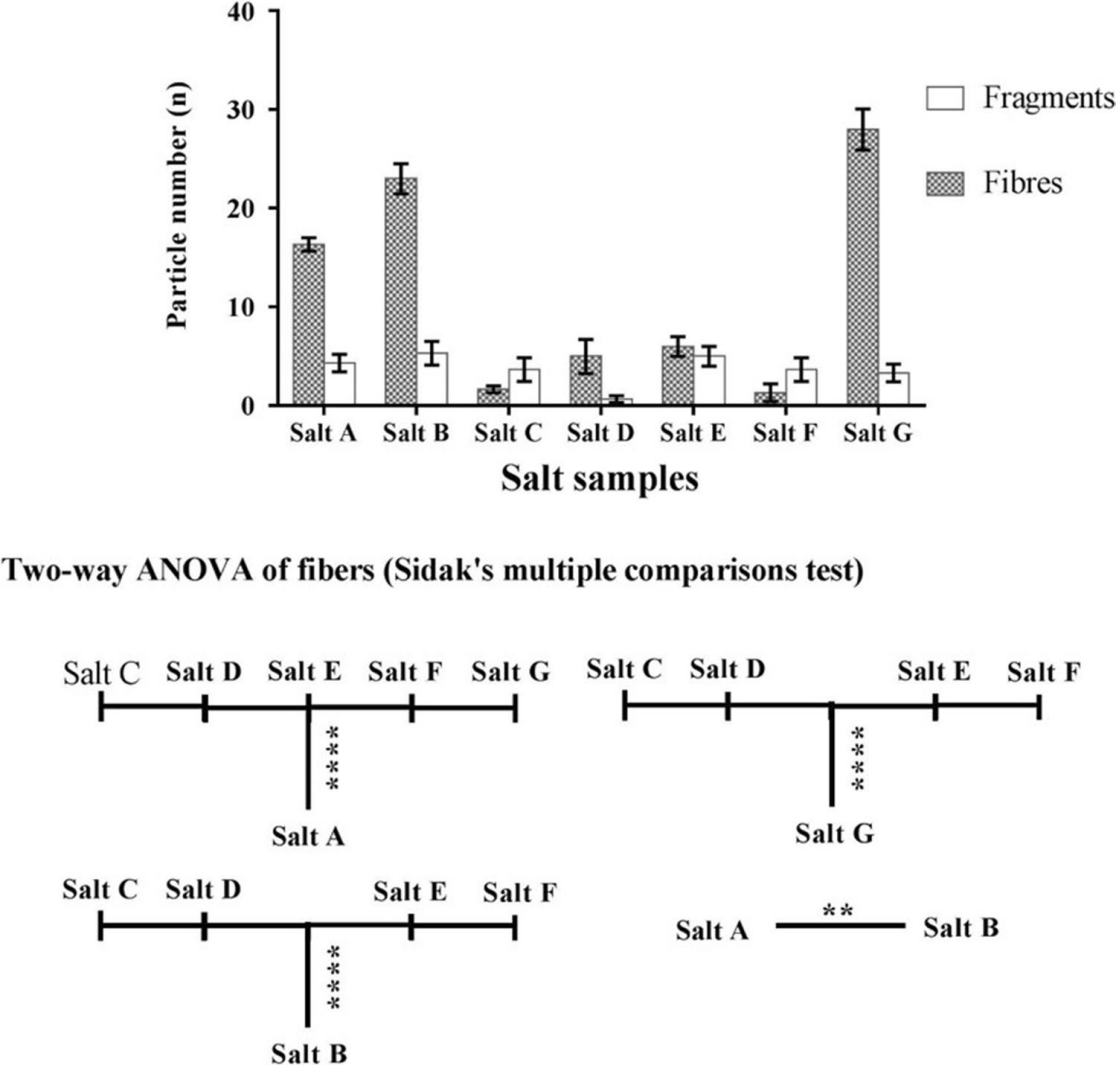
Comparison of fragments and fibres in different commercial salt samples (in 180 g of salt sample). Adjusted *p* value: ****p < 0.0001, **p = 0.0078. Fibre number in salt A was statistically significant to fibre number in salts C, D, E, and F (*p* < 0.0001). Fibres in salt G were statistically significant with fibres in salts C, D, E, and F (*p* < 0.0001). Similarly, fibre number in salt B was statistically significant with fibres in salts C, D, E, and F (*p* < 0.0001). Finally, fibres in salt A were statistically significant with fibre number in salt B (*p* = 0.0078)

Two-way ANOVA using Sidak multiple comparison tests showed statistically significant MP abundance in a few salt samples (Fig. 4). The HPC sample had the highest number (34.43%) of fibres among all samples. Another salt sample HPF (fine Himalayan salt) was analysed from the same origin (i.e. Salt Mines, Pakistan) to verify accidental contamination of fibres during the manufacturing process in HPC. HPF had very little (1.63%) fibre concentration, although fragments from HPF contributed considerably more to the overall fragment number (14.10%). The comparative difference in the number of MPs from the salts of the same origin might be due to differences in the industrial or manufacturing setups. Hence, it is likely that MP contamination might have occurred during the manufacturing process, adding up to existing contamination. Due to their small size, most fibres found in HPF and HPC surpassed the ATR-FTIR detection limit of < 20 µm. The second highest fibre number was seen in BS (28.27%), and the lowest fragment concentration was seen in IS (2.56%). Further, the size of isolated MPs among all the samples ranged from 23.2 µm to 3.9 mm. Fibres and fragments were the dominant shapes of MPs identified in the study. A statistically significant difference was seen in fibre number among the salt samples (Fig. 4). Due to size limitations, few transparent sheets were observed in the isolated MPs; however, they were not considered for further analysis using ATR-FTIR.

### Identification and characterisation of MPs

**Fig. 5:**
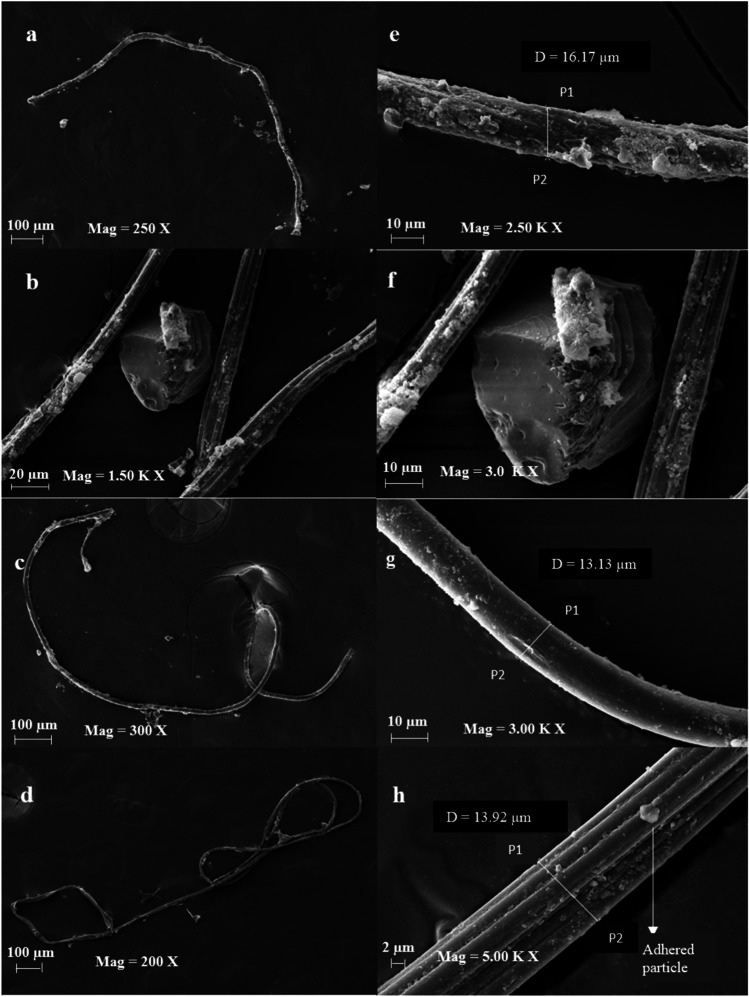
SEM images of isolated particles from salt samples. **a**, **c**, **d** Fibres and **b** fragments. **e**–**h** depict the surface adhered particles on **a**–**d**.* D* is the width of fibres (i.e. distance between P1 to P2)

**Fig. 6:**
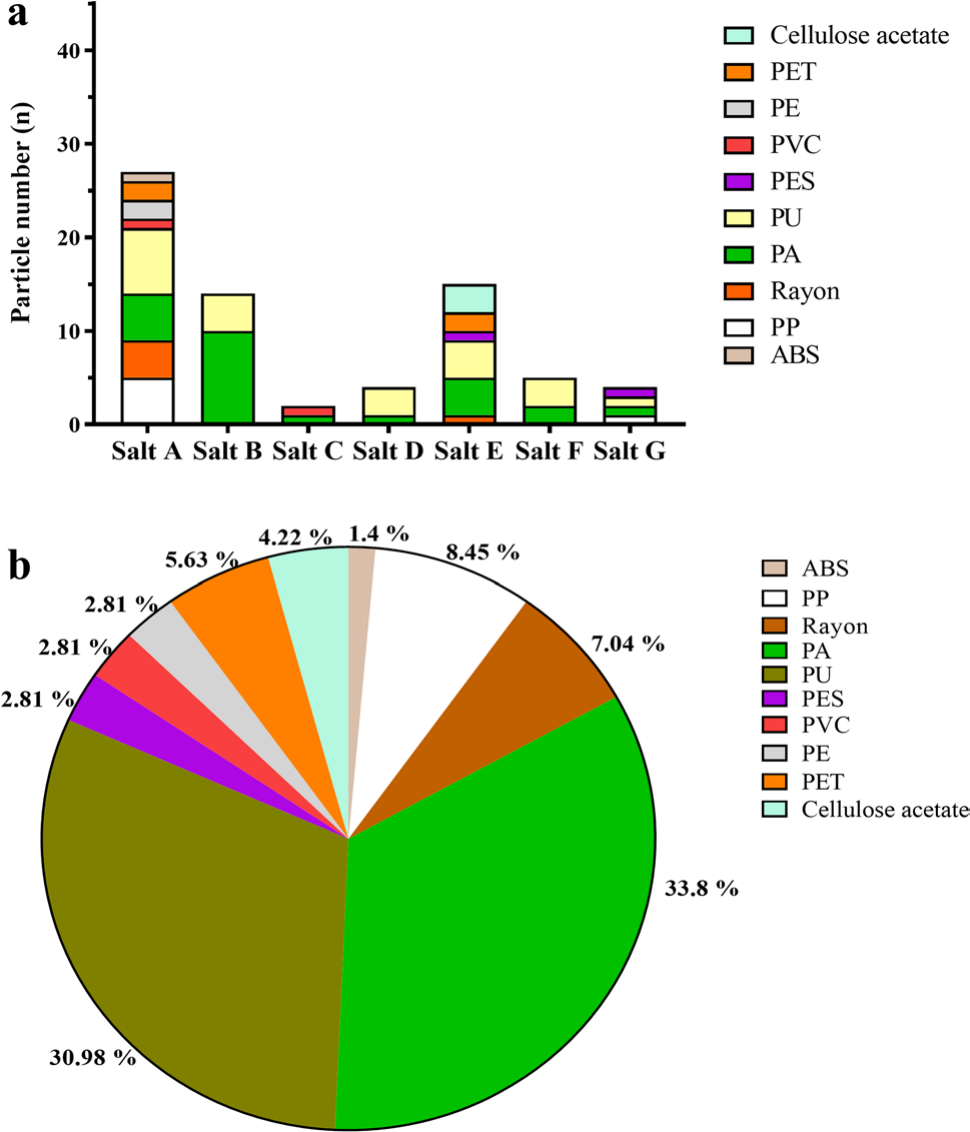
**a** Polymer chemistry of isolated microplastics from commercial salts. **b** Proportion of identified polymer types

**Fig. 7:**
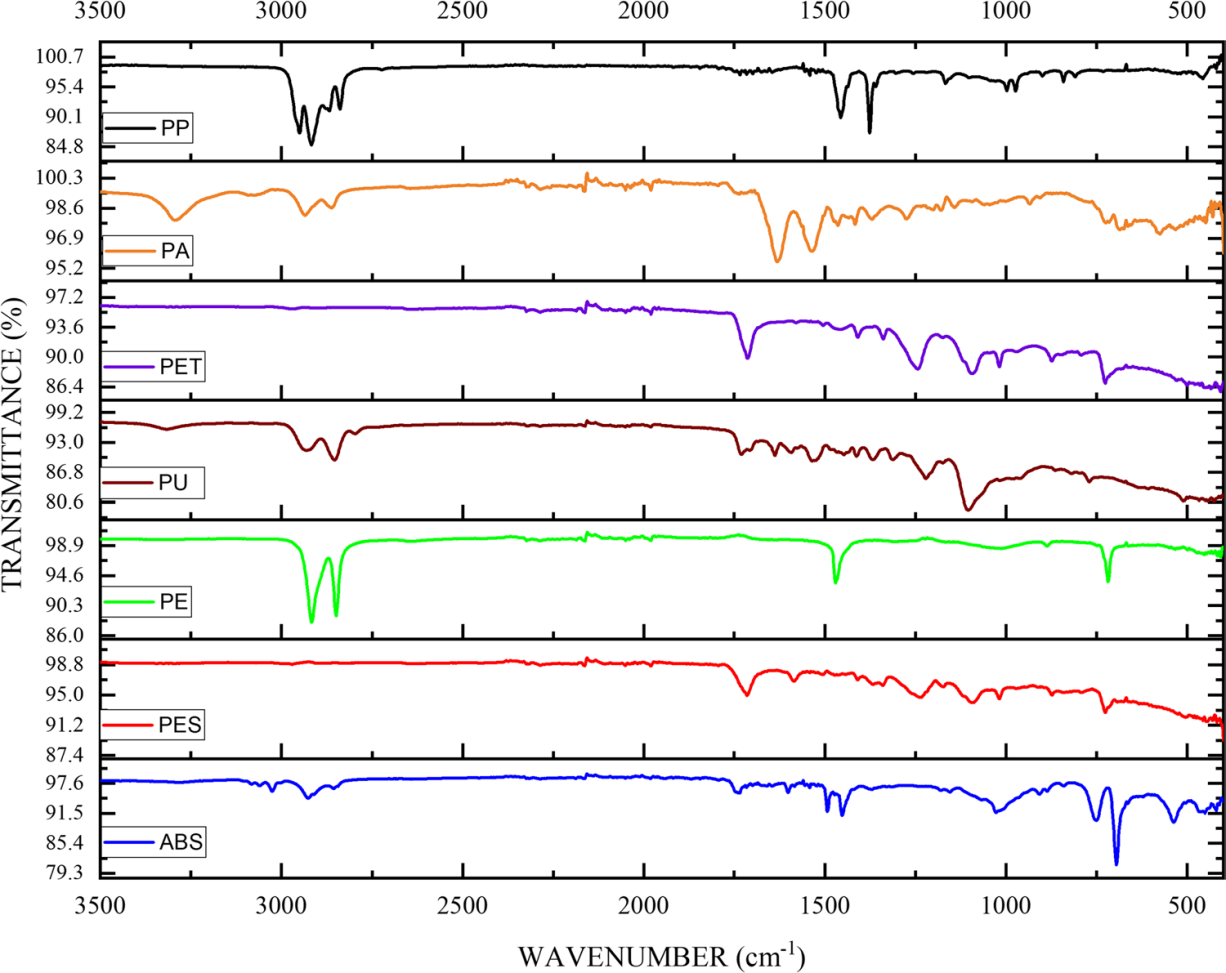
ATR-FTIR spectral data of the microplastics found in the salt samples

The scanning electron microscopy generated high-resolution images of MP’s surface state and provided an understanding of their morphological structure (Fig. 5). Elements such as carbon (C), oxygen (O), silicon (Si), chlorine (Cl), iron (Fe), iodine (I), calcium (C), magnesium (Mg), and potassium (K) were identified using EDS (Fig. S2). The elemental analysis of fibres exhibited a strong C and O peak, suggesting the possibility of plastic fibres. However, natural fibres can also have a similar structure and C signal (Remy et al. 2015), and SEM–EDS cannot differentiate between natural and plastic fibres. Therefore, a total of 90 suspected MPs were selected and identified using ATR-FTIR, out of which 71 were confirmed as MPs by matching their respective spectra (Fig. S3) with the Bruker polymer library (version 2004). The proportion of total identified suspected MPs is 78.88%. The MP composition identified were polyethylene terephthalate (PET, 5.63%), polyester (PES, 2.81%), polyethylene (PE, 2.81%), polypropylene (PP, 8.45%), cellulose acetate (4.22%), rayon (7.04%), polyurethane (PU, 30.98%), polyamide (PA, 33.8%), polyvinylchloride (PVC, 2.81%), and acrylonitrile butadiene styrene (ABS, 1.4%) (Fig. 6). The ATR-FTIR spectrums of abundant MPs are represented in Fig. 7. Glass fibres were only observed in IS. From the 71 identified MPs, fibres and fragments contributed 75.78% and 24.22%, respectively. The dominant MPs in TS were PU (31.8%), followed by PA (20.8%) and PP (83.3%) from the overall analysed particle. Moreover, PE, PET, and rayon were also detected in TS. The most dominant MP type in BS was PA (41.6%) followed by PU (18.1%). Overall, ATR-FTIR analysis followed by a Bruker polymer library search confirmed that PA (33.8%) and PU (30.98%) were the dominant MP. Cellulose acetate (20%), commonly used in mixtures with synthetic fibres in textile industries, was detected in the RS samples. The SS, a product from the great barrier reef in Australia, contained PVC (50%) and PA (4.16%), while most PA in samples were fibres.

## Discussion

In the present study, we used light microscopy and ATR-FTIR technologies to identify and characterise the MPs from different commercial edible salts available in the Australian market. The methodology is well accepted and widely utilised for microplastic research (Yang et al. 2015). For FTIR analysis, isolated particles from the triplicates were pooled into respective salt samples. The particles were then selected in varying sizes (> 15 µm) from the pooled samples for ATR-FTIR analysis. Two particles (in the size range of 15 to 20 µm) each were selected from HPC and HPF; however, these particles did not produce any spectrum. Therefore, we quantified particles > 20 µm. The obtained spectral data from various suspected particles was matched with the Bruker polymer library to identify the polymer chemistry. However, considering the complexity of environmental samples, the match quality index was not high, likely to result from staining with Nile red staining performed to visualise the particles. In addition, other factors like environmental degradation, weathering, and insufficient particle numbers might also contribute to a low-quality index (Woodall et al. 2014).

The mean MP concentration in the present study ranged from 27.78 ± 14.70 to 174.07 ± 25.05 particles/kg. The number of MPs in the present study was compared with MPs’ concentration in previous studies. Firstly, a study conducted with 15 brands of different Chinese salts reported 7–204 particles/kg in rock/well salts, 550–681 particles/kg in sea salts, and 43–364 particles/kg in lake salts (Yang et al. 2015). Likewise, an investigation of 8 types of sea salts in India by Seth et al. (2018) corresponded to 56 ± 49 to 103 ± 39 particles/kg of MP contamination (Seth and Shriwastav 2018). Conversely, in the present study, the MP pollution in sea salt was lower (29.63 ± 13.98 particles/kg) than in Chinese and Indian salts. Such difference might be due to high MP contamination in the Chinese and Indian seas compared with the Australian sea. Iniguez et al. (2018) investigated 21 commercial brands of salts and reported the presence of MPs in Spanish tablet salt ranging from 50 to 280 MPs/kg (Iñiguez et al. 2017). The present investigation observes 114.81 ± 13.98 particles/kg in the Australian table salt. Few previous studies reported considerably low mean MP contaminations in salts (Karami et al. 2017; Lee et al. 2019). In Taiwan, Lee et al. (2019) investigated the presence of MPs in 11 different salts and detected average contamination of 9.77 particles/kg of salts (Lee et al. 2019). Karami et al. (2017) investigated the presence and concentration of MPs in 17 different brands of salt across eight countries (Australia, Iran, Japan, Malaysia, New Zealand, France, Portugal, and South Africa). MPs were detected in 88% of the salts analysed, and particle concentrations ranged from 0 (French sea salt) to 10 particles/kg (Portuguese sea salt) in the salt samples (Karami et al. 2017). From the 11 studies conducted around the world on salt MPs, our global review concluded that PP was highly abundant in European and Asian salts, PE in Oceanian salts, PE and PET in South American salts, PE in North American salts, and PET in African salts (Table S2, Figs. S4, S5, and S6) (Fadare et al. 2021; Gündoğdu 2018; Iñiguez et al. 2017; Karami et al. 2017; Kim et al. 2018; Kosuth et al. 2018; Lee et al. 2019; Renzi and Blašković 2018; Sathish et al. 2020; Seth and Shriwastav 2018; Yang et al. 2015). A global map representing the contamination of MPs in salt per kg was generated (Fig. S5).

SEM–EDS characterisation aided in identifying the adsorbed debris on the surface of MPs. Most of the reported elements such as Ca, Mg, and Si in this study are antioxidants that are added to aid in slowing down the oxidation in polymers such as PE, PP, and PS (Dehghani et al. 2017). The presence of Cl elements may be an indication of adhered chloride ions from salt on the MPs’ surfaces. The significant peak of adhered iodine on the fibres can be correlated with the use and addition of iodine in iodised salts for reducing iodine deficiency. Thus, the particles isolated from iodised salts may have incorporated this iodine onto their surfaces. Even though SEM–EDS is widely used to assess morphology and elemental composition, it is a time-consuming and costly technology. Furthermore, since the isolation of MPs is dependent on the researcher’s expertise, chemical characterisation may be prone to a selection bias (Blair et al. 2019).

Previous studies on MPs in Australia’s commercial salts show the presence of PE, PET, acrylic, nylon, PET, PP, and PS (Karami et al. 2017; Kim et al. 2018). However, our study also reports the existence of PU and PVC in Australian commercial salts. The present study interprets the abundance of PA and PU, which is different from previous reports and relates to the selection of brands/samples. Previous studies had focused on the sea, lake, and rock salts, but in the present study, the inclusion of BS with high PA is an additional knowledge. PA-66 (also known as nylon 6,6) was identified in the samples and was categorised in PA. Likewise, PA MPs were also reported in the previous studies on Indian salt (Sathish et al. 2020; Seth and Shriwastav 2018). The possible sources of PA fibres may be due to extensive fishing activity and accidental or intentional release of fishing gear and nets into seas/oceans (Castelvetro et al. 2021). Other possible sources include heavy-duty tyres, industrial fabrics, and light or highly durable ropes used in the industries (Hearle 2001). Also, PA is used in making car handles, seat belt components, stitches, airbag containers, pedals, etc. and has replaced the metal in-seat systems (Palmer 2001). Due to the exclusive properties of PU such as elongation, strength, and hardness, this material is widely used in building and constructions, automotive, textiles (Chattopadhyay and Raju 2007; Zia et al. 2014), cushion materials, carpet underlays, bedding, furniture, shoe soles, etc. (Cinelli et al. 2013). Therefore, the possibility of airborne PU particles originating from beddings, furniture, shoe soles, clothes of workers, etc. in the industries and contaminating the products cannot be neglected.

PP (8.45%), rayon (7.04%), and PET (5.63%) were abundant polymer types after PA and PU in analysed salt samples. The abundance of PP can be attributed to low density (0.90–0.91 g/cm^3^), facilitating their distribution by becoming airborne and also allowing them to float on the water surfaces in the saltpans (Karami et al. 2017). Rayon, an artificial non-plastic polymer, contributed 56.9% of all the sediment and coral samples analysed in the Indian ocean (Woodall et al. 2014). We have reported rayon in the MP’s research, as it is man-made, used in clothing, personal hygiene products, and cigarette filters, and can either be introduced into the marine environment through sewage (Woodall et al. 2014) or by the release of fibres from clothing directly to air (De Falco et al. 2020). As clothes are one of the sources of rayon fibres, these may get airborne directly or during washing (De Falco et al. 2020) and can contaminate the salt samples in an industry setting, although further investigations should be done specifically to reveal the possibilities of such a claim. ABS in salt samples may have been incorporated from the wear and tear of tyres of automobiles in the industries. Another source of ABS in industries is the belts in conveyor belts. The belt in the conveyor belts are exposed to heavy loads, and this permanent or temporary contact between load and belt surfaces leads to the release of wear particles (Andrejiova et al. 2016; Bindzár 2002). The data on the release of fibres or wear particles from the conveyor belts are scarce and further study should be done to extrapolate the extent of MP release from conveyor belts. The most abundant polymer reported in Chinese salts was PET, likely to be due to its high density (1.30 g/cm^3^) (Yang et al. 2015). The predominant types of MPs in Spanish table salt were also PET (83.3%), followed by PP (6.7%) and PE (3.3%). Conversely, PET only contributed to 5.63% of all MPs analysed in our study. PET MPs were also reported from the previous salt investigation in Australia (Karami et al. 2017; Kim et al. 2018).

The Himalayan rock salts are commonly extracted through mining, where chances of atmospheric MP deposition are less; however, the observed rise in MP contamination in the mined salt is likely due to the addition of airborne MPs during the processing and packaging of salts. The information on the packaging, sources, and type were provided in the labelling of the salt samples. However, no information on the type was indicated on the labels of salt A and salt D. In the present study, salt A was packed on hard paper, while salts B, C, E, and F in PE, and salts D and G in high-density PE. Polyethylene MPs from the packaging material may have broken down and influenced the concentration of isolated PE MPs in salt, although it is unclear how much amount of the broken particles from packaging may have contributed to the overall PE proportion in the present study. Thus, a detailed study to track the amount of contribution of packaging material in the commercial salt is required. The BS is commonly produced in India and Pakistan through mining, and the raw materials required for the production of BS are sometimes taken from the North Indian salt lakes such as Sambhar Lake (Chandrashekhar 1977). An investigation of the quantity of MP contamination in these lakes may provide an insight into the MP contamination in the raw materials of BS alone. Limited studies in these lakes provide scope for future investigation of MPs. Globally, it is clear that MP in salt is a major concern to human health; however, limited studies in continents like North and South America are challenging to anticipate the MP incidence in each continent with efficiency. Therefore, industries producing salts may be recommended to take consecutive measures and initiate strategies to reduce or eliminate the MPs during manufacturing and packaging processes.

According to Land et al. (2018), daily salt consumption in Australian adults is greater than the maximum recommended figure by WHO (i.e. 5 g). Australian men’s and women’s salt consumption are 10.1 g and 7.34 g per day respectively (Land et al. 2018). Therefore, considering an average MP contamination at 85.19 ± 63.04 (SD) particles/kg, MP ingestion in Australian men and women would be calculated approximately at 314.05 and 228.23 particles/year respectively. The limitations of these studies include the following: (1) sample size for salts analysed is low and an increase in salt samples would have pointed to efficient data in terms of yearly intake of MPs from salt, and (2) salts of different varieties were selected with no information on consumer preference, and the study assumes that the selected salts are purchased frequently.

## Conclusion

The present study concluded that MP contamination in Australian commercial salts ranged from 27.78 ± 14.70 to 174.07 ± 25.05 particles/kg (mean: 85.19 ± 63.04 particles/kg). According to the World Health Organization (WHO), adults consume less than 5 g of salts daily. Considering an adult consuming a maximum of 5 g, on average, the Australian population is ingesting approx. 155.47 particles per year. However, these figures may raise based on reports on higher salt consumption by Australians than that recommended by WHO. Thus, MP ingestion by Australian men and women would be approximately 314.05 and 228.23 particles/year respectively. MP contamination in terrestrial salts such as black salts and Himalayan pink salt were higher compared to marine salts. The study also shows that the MP contamination in mined salts would have arisen from the manufacturing process or packaging and storage operations. The role of airborne MPs by their atmospheric deposition in salt contamination cannot be neglected as the particle number of isolated fibres is considerably alarming. Salt is one of the significant ingredients in food, and therefore, strategies for reducing or eliminating the MP contamination in commercial salts are crucial for a better and healthier life.

## Supplementary Information

Below is the link to the electronic supplementary material.

Supplementary file1 (DOCX 66 KB)

Supplementary file2 (DOCX 5009 KB)

## Data Availability

The datasets used and/or analysed during the current study are available from the authors on reasonable request.
