# Supplementary material for: Consuming microplastics? Investigation of commercial salts as a source of microplastics (MPs) in diet
Source: Environ Sci Pollut Res Int. 2022 Jul 30;30(1):930–42. doi: 10.1007/s11356-022-22101-0 (PMC9813175; doi:10.1007/s11356-022-22101-0)
Supplement: Supplementary file 1 — Supplementary file1 (DOCX 66 KB) [file 11356_2022_22101_MOESM1_ESM.docx]

**Consuming Microplastics? Investigation of commercial salts as a source of microplastics (MPs) in diet**

Aswin kuttykattil^1^, [Subash Raju](https://www.researchgate.net/profile/Nsubash-Raju)^1^, Kanth Swaroop Vanka^1,3^, Geetika Bhagwat^1^, Maddison Carbery^1^, Salom Gnana Thanga Vincent ^1,4^, Sudhakaran Raja^2^, Thava Palanisami^1*^.

*1.* *Environmental Plastics Innovation Cluster, Global Innovative Centre for Advanced Nanomaterial, The University of Newcastle, Newcastle, NSW, Australia*

*2. Aquaculture Biotechnology Laboratory, School of Bio-Sciences and Technology, Vellore Institute of Technology, Vellore, India*

*3. School of Biomedical Sciences and Pharmacy, The University of Newcastle/ Priority Research Centre for Healthy Lungs, Hunter Medical Research Institute, The University of Newcastle, Newcastle, NSW, Australia*

*4. Department of Environmental Sciences, University of Kerala, Kerala, India*

***Corresponding Author**: Dr. Thava Palanisami, Environmental Plastics Innovation Cluster (EPIC) , Global Innovative Centre for Advanced Nanomaterials (GICAN), The University of Newcastle, Callaghan, NSW, 2308, Australia.

Email: [thava.newcastle@gmail.com](mailto:thava.newcastle@gmail.com)

**Table S1**: Colour classification of MPs found in salt samples

**Batch 1:**

|  | **Fragments** | | | | | | | | | | **Fibre** | | | | | | | | | **Fragment + Fibre** |
| --- | --- | --- | --- | --- | --- | --- | --- | --- | --- | --- | --- | --- | --- | --- | --- | --- | --- | --- | --- | --- |
| **Samples** | **Blue** | **Blac-k** | **Green** | **Orange** | **Red** | **White** | **violet** | **Pink stained** | **Clear** | **Subtotal** | **Blue** | **Black** | **Green** | **Orange** | **Red** | **White** | **Pink stained** | **Clear** | **Subtotal** | **TOTAL** |
| **TS** | 1 | 0 | 0 | 1 | 0 | 0 | 0 | 2 | 2 | **6** | 0 | 7 | 0 | 0 | 0 | 9 | 0 | 1 | **17** | **23** |
| **SS** | 0 | 0 | 0 | 0 | 0 | 5 | 0 | 1 | 0 | **6** | 0 | 1 | 0 | 0 | 0 | 0 | 1 | 0 | **2** | **8** |
| **HPF** | 0 | 1 | 0 | 0 | 0 | 0 | 0 | 2 | 0 | **3** | 0 | 3 | 0 | 0 | 0 | 0 | 0 | 0 | **3** | **6** |
| **RS** | 0 | 0 | 0 | 0 | 0 | 1 | 0 | 2 | 0 | **3** | 2 | 0 | 0 | 0 | 0 | 1 | 1 | 0 | **4** | **7** |
| **BS** | 0 | 0 | 0 | 0 | 0 | 3 | 0 | 0 | 0 | **3** | 7 | 6 | 0 | 0 | 2 | 6 | 1 | 0 | **22** | **25** |
| **IS** | 0 | 0 | 0 | 0 | 0 | 0 | 0 | 0 | 0 | **0** | 0 | 4 | 0 | 0 | 0 | 0 | 1 | 0 | **5** | **5** |
| **HPC** | 0 | 0 | 0 | 0 | 0 | 0 | 0 | 1 | 1 | **2** | 5 | 8 | 0 | 1 | 0 | 0 | 2 | 13 | **29** | **31** |
| **Subtotal** | **1** | **1** | **0** | **1** | **0** | **9** | **0** | **8** | **3** | **23** | **14** | **29** | **0** | **1** | **2** | **16** | **6** | **14** | **82** | **105** |
| **Average** | 0.14 | 0.29 | 0.00 | 0.14 | 0.0 | 2.57 | 0.00 | 2.00 | 0.57 | 5.71 | 4.00 | 7.29 | 0.00 | 0.33 | 0.57 | 3.29 | 1.71 | 3.86 | 21.00 | 26.71 |
| **Standard deviation** | 0.37796 | 0.37796 | 0 | 0.37796 | 0 | 1.97605 | 0 | 0.89974 | 0.7868 | 2.13809 | 2.88675 | 3.02372 | 0 | 0.40825 | 0.75593 | 3.68394 | 0.69007 | 4.86484 | 10.8584 | 10.90871 |
| **Standard Error** | 0.14 | 0.14 | 0.00 | 0.14 | 0.00 | 0.75 | 0.00 | 0.34 | 0.30 | 0.81 | 1.09 | 1.14 | 0.00 | 0.15 | 0.29 | 1.39 | 0.26 | 1.84 | 4.10 | 4.12 |

**Batch 2:**

|  | **Fragments** | | | | | | | | | | **Fibre** | | | | | | | | | **Fragment + Fibre** |
| --- | --- | --- | --- | --- | --- | --- | --- | --- | --- | --- | --- | --- | --- | --- | --- | --- | --- | --- | --- | --- |
| **Sample no.** | Blue | black | Green | Orange | Red | White | violet | Pink stained | Clear | **Subtotal** | Blue | Black | Green | Orange | Red | White | Pink stained | Clear | Subtotal | **TOTAL** |
| **TS** | 0 | 0 | 0 | 0 | 0 | 2 | 0 | 2 | 0 | **4** | 0 | 7 | 0 | 0 | 1 | 7 | 1 | 1 | 17 | **21** |
| **SS** | 0 | 0 | 0 | 0 | 0 | 3 | 0 | 0 | 0 | **3** | 0 | 0 | 0 | 0 | 0 | 0 | 2 | 0 | 2 | **5** |
| **HPF** | 0 | 2 | 0 | 0 | 0 | 0 | 0 | 4 | 0 | **6** | 0 | 1 | 0 | 0 | 0 | 0 | 0 | 0 | 1 | **7** |
| **RS** | 1 | 0 | 0 | 0 | 0 | 2 | 0 | 2 | 1 | **6** | 0 | 3 | 0 | 0 | 0 | 2 | 0 | 2 | 7 | **13** |
| **BS** | 1 | 0 | 0 | 1 | 0 | 3 | 0 | 2 | 0 | **7** | 0 | 16 | 0 |  | 6 | 4 | 0 | 0 | 26 | **33** |
| **IS** | 0 | 0 | 0 | 0 | 0 | 0 | 0 | 1 | 0 | **1** | 2 | 5 | 0 | 0 | 0 | 0 | 1 | 0 | 8 | **9** |
| **HPC** | 1 | 0 | 0 | 0 | 0 | 2 | 0 | 2 | 0 | **5** | 6 | 7 | 0 | 0 | 0 | 1 | 0 | 17 | 31 | **36** |
| **Subtotal** | **3** | **2** | **0** | **1** | **0** | **12** | **0** | **13** | **1** | **32** | **8** | **39** | **0** | **0** | **7** | **14** | **4** | **20** | **92** | **124** |
| **Average** | 0.86 | 0.57 | 0.00 | 0.29 | 0.00 | 3.14 | 0.00 | 3.43 | 0.29 | **8.57** | 2.29 | 10.14 | 0.00 | 0.00 | 1.86 | 3.00 | 1.00 | 5.57 | 23.86 | **32.43** |
| **Standard deviation** | 0.53452 | 0.75593 | 0 | 0.37796 | 0 | 1.25357 | 0 | 1.21499 | 0.37796 | **2.070197** | 2.26779 | 5.34968 | 0 | 0 | 2.23607 | 2.64575 | 0.7868 | 6.28301 | 11.79588 | **12.60574** |
| **Standard Error** | **0.20** | **0.29** | **0.00** | **0.14** | **0.00** | **0.47** | **0.00** | **0.46** | **0.14** | **0.78** | **0.86** | **2.02** | **0.00** | **0.00** | **0.85** | **1.00** | **0.30** | **2.37** | **4.46** | **4.76** |

**Batch 3:**

|  | **Fragments** | | | | | | | | | | **fibre** | | | | | | | | | **Fragment + Fibre** |
| --- | --- | --- | --- | --- | --- | --- | --- | --- | --- | --- | --- | --- | --- | --- | --- | --- | --- | --- | --- | --- |
| **Sample no.** | **Blue** | **black** | **Green** | **Orange** | **Red** | **White** | **violet** | **Pink stained** | **Clear** | **Subtotal** | **Blue** | **Black** | **Green** | **Orange** | **Red** | **White** | **Pink stained** | **Clear** | **Subtotal** | **TOTAL** |
| **TS** | 0 | 0 | 0 | 1 | 0 | 1 | 0 | 1 | 0 | **3** | 0 | 5 | 0 | 0 | 1 | 4 | 0 | 5 | **15** | **18** |
| **SS** | 0 | 0 | 0 | 0 | 0 | 2 | 0 | 0 | 0 | **2** | 0 | 0 | 0 | 0 | 0 | 0 | 1 | 0 | **1** | **3** |
| **HPF** | 0 | 0 | 0 | 0 | 0 | 0 | 0 | 2 | 0 | **2** | 0 | 0 | 0 | 0 | 0 | 0 | 0 | 0 | **0** | **2** |
| **RS** | 1 | 0 | 0 | 0 | 0 | 2 | 0 | 2 | 1 | **6** | 0 | 3 | 0 | 0 | 0 | 2 | 0 | 2 | **7** | **13** |
| **BS** | 2 | 0 | 0 | 0 | 0 | 2 | 0 | 2 | 0 | **6** | 0 | 11 | 0 | 0 | 6 | 4 | 0 | 0 | **21** | **27** |
| **IS** | 0 | 0 | 0 | 0 | 0 | 0 | 0 | 1 | 0 | **1** | 0 | 2 | 0 | 0 | 0 | 0 | 0 | 0 | **2** | **3** |
| **HPC** | 0 | 0 | 0 | 0 | 0 | 1 | 0 | 2 | 0 | **3** | 6 | 7 | 0 | 0 | 0 | 1 | 0 | 10 | **24** | **27** |
| **Subtotal** | **3** | **0** | **0** | **1** | **0** | **8** | **0** | **10** | **1** | **23** | **6** | **28** | **0** | **0** | **7** | **11** | **1** | **17** | **70** | **93** |
| **Average** | 0.86 | 0.00 | 0.00 | 0.14 | 0.0 | 2.14 | 0.00 | 2.71 | 0.29 | 6.14 | 1.71 | 7.29 | 0.00 | 0.00 | 1.86 | 2.57 | 0.29 | 4.14 | 17.86 | 24.00 |
| **Standard deviation** | 0.7868 | 0 | 0 | 0.37796 | 0 | 0.89974 | 0 | 0.7868 | 0.37796 | 1.976047 | 2.26779 | 4 | 0 | 0 | 2.23607 | 1.81265 | 0.37796 | 3.82349 | 9.966611 | 11.08624 |
| **Standard Error** | 0.30 | 0.00 | 0.00 | 0.14 | 0.0 | 0.34 | 0.00 | 0.30 | 0.14 | 0.75 | 0.86 | 1.51 | 0.00 | 0.00 | 0.85 | 0.69 | 0.14 | 1.45 | 3.77 | 4.19 |

**Table S2**: Global data on the type of salts, size range, number of particles/kg, chemical composition, and shapes of microplastics. (modified and updated table from (Diogo Peixot, 2019))

| **S.no** | **Continent** | **Countries** | **Type of salts** | **Size range**  **(μm)** | **Number of particles (Particles.kg^-1^)** | **Chemical composition of MPs** | **Shapes of microplastics** | **References** |
| --- | --- | --- | --- | --- | --- | --- | --- | --- |
| 1 | Europe | 1. Portugal 2. France 3. Spain 4. United Kingdom 5. Bulgaria 6. Croatia 7. Italy 8. Belarus 9. Germany 10. Hungary | Sea salt  Sea salt  Sea salt    Sea salt  Sea salt(F)  Sea salt(C)  Well salt  Sea salt  Sea salt  Sea salt  Sea salt  Rock salt  Rock salt  Rock salt  Rock salt | 160-980  160-980  160-980  30-3500  30–3500  30–3500  30–3500  100-2000  100-4000  15-4628  4–2100  100–2000  100–5000  0-100  100–4000 | 0-10  1-3  0-2  50-150  80–280  60–65  120–185  120  10  13500–19800  22–594  80  8  2  12 | PET and PP  PE, PET and PP  PE, PET and PP  PE, PET and PP  PE, PET and PP  PE, PET and PP  PE, PET and PP  PP, PE and PVC  PE, PP, PVC and Nylon  PE and PP  PE and PP  PE, PET, PP and PVC  PET  PET  Nylon, PET, PR and PVC | Fragments, Films and Filament  Fragments, Films and Filament  Fragments, Films and Filament  Fibre  Fibre  Fibre  Fibre  Fragment, fibre and sheet  Fragment, fibre and sheet  Fibre, fragment, granules and films  Fibre, fragment, granules and films  Fragment, fibre and sheet  Fragment, fibre and sheet  Fragment, fibre and sheet  Fragment, fibre and sheet | (Karami et al., 2017)  (Karami et al., 2017)  (Karami et al., 2017)  (Iñiguez et al., 2017)  (Iñiguez et al., 2017)  (Iñiguez et al., 2017)  (Iñiguez et al., 2017)  (Kim et al., 2018)  (Kim et al., 2018)  (Renzi and Blašković, 2018)  (Renzi and Blašković, 2018)  (Kim et al., 2018)  (Kim et al., 2018)  (Kim et al., 2018)  (Kim et al., 2018) |
| 2 | Asia | 1. Turkey 2. India 3. Malaysia 4. China 5. Chinese   Taipei   1. Indonesia 2. Japan 3. Korea 4. Thailand 5. Vietnam 6. Pakistan 7. Iran 8. Philippines 9. Taiwan | Sea salt  Lake salt  Rock salt  Sea salt  Sea salt  Sea Salt  Sea salt,  Sea salt  Lake salt  Lake salt  Rock/well  salt  Rock salt  Sea salt  Sea salt  Sea salt  Sea salt  Sea salt  Sea salt  Rock salt (Himalayan pink salt)  Rock salt  Lake salt  Rock salt  Sea Salt | <100 - >1000  <100 -  >1000  200 - >1000  500–2000  100-5000  100-1000  160-980  <100 -  >1,000  100–4000  <100 -  >1,000  100–2000  <100 -  >1000  0–4000  100–5000  100–2000  160-980  100–3000  100–5000  100–5000  40–5000  100–1000  160–980  100–5000  1 -1500 | 18-84  8–102  9–16  56-103  50-600  2-72  0-1  550–681  20–718  43–364  28  7–204  0–14  0–1300  100  0-1  100–300  80–600  100–200  367  100  0–1  120  9.77 | PE, PP, PU, PVC, PET and PA-6  PE, PET, PP, PU, PMA, PA-6 and PVC  PP  PA, PE, PET, PS and PES  Nylon, PE, PET, PP and PVC  PE, PES, PP and PA  PP  PP , CP, CL, PAN, PB, PE, PES, POM, PMA and PET  Nylon, EVA, PE, PET, PP, PU and PVC  PET, PE-PP, PES, PB, PP, POM, PAK and CP  PE, PET, PP, PS and Teflon  PET, PE, PB, PP, PE-PP, POM, PAN, PVC, EVA, CP and CL  PET, PP and Teflon  Acrylic, Nylon, PE, PET, PP, PVC and PW  PE, PET and PP  PE and PET  Acrylic, Nylon, PE, PET and PP  PE, PET, PP and PVC  Acrylic, PE, PP and PW    -  EVA, PE, PET and PP  PP  PE, PET, PP and PVC  PET, PP, PES, PEI, PE and PAC | Fragment, fibre and film  Fragment, fibre and film  Fragment, fibre and film  Fragment and fibre  Fragment, fibre and sheet  Fragment and fibre  Fragment, Films and Filament  Pellet, Sheet, Fibre, Irregular fragment  Fragment (72%) and fibre (28%)  Pellet, Sheet, Fibre, Irregular fragment  Fragment, fibre and sheet  Pellet, Sheet, Fibre, Irregular fragment  Fragment, fibre and sheet  Fragment and fibre  Fragment, fibre and sheets  Fragment, fibre and filament  Fragment (90%), fibre (8.2) and sheet (2%)  Fragment, fibre and sheet  Fragment, fibre and sheet  Fibres and particles  Fragment, fibre and sheet  Fragment, fibre and filament  Fragment, fibre and sheet  Fragment and fibre | (Gündoğdu, 2018)  (Gündoğdu, 2018)  (Gündoğdu, 2018)  (Seth and Shriwastav, 2018)  (Kim et al., 2018)  (Sathish et al., 2020)  (Karami et al., 2017)  (Yang et al., 2015)  (Kim et al., 2018)  (Yang et al., 2015)  (Kim et al., 2018)  (Yang et al., 2015)  (Kim et al., 2018)  (Kim et al., 2018)  (Kim et al., 2018)  (Karami et al., 2017)  (Kim et al., 2018)  (Kim et al., 2018)  (Kim et al., 2018)  (Kosuth et al., 2018)  (Kim et al., 2018)  (Karami et al., 2017)  (Kim et al., 2018)  (Lee et al., 2019) |
| 3 | Oceania | 1. Australia 2. New Zealand | Sea salt  Sea salt  Sea salt  Sea salt | 160-980  160-980  100–3000  160–980 | 0-46  0-9  80  0–1 | PE and PET  PE and PET  Acrylic, Nylon, PE, PET, PP and PS  PE | Fragment, fibre and filament  Fragment, fibre and filament  Fragment (67%) and fibre (33%)  Fragment, fibre and filament | (Karami et al., 2017)  (Karami et al., 2017)  (Kim et al., 2018)  (Karami et al., 2017) |
| 4 | South America | 1. Brazil | Sea salt | 100-5000 | 200 | PP and PET | Fragment | (Kim et al., 2018) |
| 5 | North America | 1. USA 2. Mexico | Sea salt  Sea salt  Rock salt  Lake salt  Sea salt | 100-2000  40–5000  100  40–5000  40–5000 | 300  47  5  113  173 | PP, PE and PVC    -  PE  -  - | Fragment, Fibres and particles  Fragment, fibre and sheet  Fragment, Fibres and particles  Fibres and particles  Fibres and particles | (Kim et al., 2018)  Kosuth et al., 2018  (Kim et al., 2018)  (Kosuth et al., 2018)  (Kosuth et al., 2018) |
| 6 | Africa | 1. South Africa 2. Senegal 3. Nigeria      1. Cameroun      1. Ghana | Sea salt  Table salt  Sea salt,  Lake salt  Table salt  Table salt  Table salt | 160-980  3.3-4660  100-3000  100–4000  3.3-4660  3.3-4660  3.3-4660 | 1-3  1.33 ± 0.32  250  800  0–0.33 ± 0.38  0–0.33 ± 0.38  0–0.33 ± 0.38 | PET  PE, PP, PET, PEI and PVA  PE, PET and PP  Acrylic, PE, PET, PS and PVC  PE, PP, PET, PEI and PVA  PE, PP, PET, PEI and PVA  PE, PP, PET, PEI and PVA | Fragments, Films and fibre  Microfibers and microplastics  Fragment (62%) and fibre (38%)  Fragment and fibre and sheet  Microfibers and microplastics  Microfibers and microplastics  Microfibers and microplastics | (Karami et al., 2017)  (Fadare et al., 2021)  (Kim et al., 2018)  (Kim et al., 2018)  (Fadare et al., 2021)  (Fadare et al., 2021)  (Fadare et al., 2021) |

**Abbreviation**: Polyacetal(PAC), polyetherimide(PEI),ethylene vinyl acetate(EVA), cellulose(CL), polymethylacrylate (PMA), Polyethylene terephthalate (PET), polyethylene (PE), polypropylene (PP), cellophane (CP),Polystyrene (PS), polyester (PES),polyamide-6 (PA-6), paraffin wax (PW), phenoxy resin (PR), polyacrylate (PA), polycarbonate (PC), polyurethane (PU), polyvinylchloride (PVC), polyacrylonitrile (PAN), poly(1-butene) (PB), polymethyl methacrylate(PMMA), polyalkene (PAK),PE and PP copolymer(PE-PP), Polyetheylene isophthalate(PEI), Polyvinyl alchohol (PVA)

**REFERENCES**

Diogo Peixot C, JoãoAmorim, LuísOliva-Teles,LúciaGuilhermino, Maria Natividade Vieira,. Microplastic pollution in commercial salt for human consumption: A review. Estuarine, Coastal and Shelf Science 2019; 219: 161-168.

Fadare OO, Okoffo ED, Olasehinde EF. Microparticles and microplastics contamination in African table salts. Marine Pollution Bulletin 2021; 164: 112006.

Gündoğdu S. Contamination of table salts from Turkey with microplastics. Food Additives & Contaminants: Part A 2018; 35: 1006-1014.

Iñiguez ME, Conesa JA, Fullana A. Microplastics in Spanish table salt. Scientific reports 2017; 7: 1-7.

Karami A, Golieskardi A, Choo CK, Larat V, Galloway TS, Salamatinia B. The presence of microplastics in commercial salts from different countries. Scientific Reports 2017; 7: 46173.

Kim J-S, Lee H-J, Kim S-K, Kim H-J. Global Pattern of Microplastics (MPs) in Commercial Food-Grade Salts: Sea Salt as an Indicator of Seawater MP Pollution. Environmental science & technology 2018; 52: 12819-12828.

Kosuth M, Mason SA, Wattenberg EV. Anthropogenic contamination of tap water, beer, and sea salt. PloS one 2018; 13: e0194970.

Lee H, Kunz A, Shim WJ, Walther BA. Microplastic contamination of table salts from Taiwan, including a global review. Scientific Reports 2019; 9: 10145.

Renzi M, Blašković A. Litter & microplastics features in table salts from marine origin: Italian versus Croatian brands. Marine Pollution Bulletin 2018; 135: 62-68.

Sathish MN, Jeyasanta I, Patterson J. Microplastics in Salt of Tuticorin, Southeast Coast of India. Archives of Environmental Contamination and Toxicology 2020; 79: 111-121.

Seth CK, Shriwastav A. Contamination of Indian sea salts with microplastics and a potential prevention strategy. Environmental Science and Pollution Research 2018; 25: 30122-30131.

Yang D, Shi H, Li L, Li J, Jabeen K, Kolandhasamy P. Microplastic Pollution in Table Salts from China. Environ Sci Technol 2015; 49: 13622-7.
