# Supplementary material for: Consuming microplastics? Investigation of commercial salts as a source of microplastics (MPs) in diet
Source: Environ Sci Pollut Res Int. 2022 Jul 30;30(1):930–42. doi: 10.1007/s11356-022-22101-0 (PMC9813175; doi:10.1007/s11356-022-22101-0)
Supplement: Supplementary file 2 — Supplementary file2 (DOCX 5009 KB) [file 11356_2022_22101_MOESM2_ESM.docx]

**Consuming Microplastics? Investigation of commercial salts as a source of microplastics (MPs) in diet**

Aswin kuttykattil^1^, [Subash Raju](https://www.researchgate.net/profile/Nsubash-Raju)^1^, Kanth Swaroop Vanka^1,3^, Geetika Bhagwat^1^, Maddison Carbery^1^, Salom Gnana Thanga Vincent ^1,4^, Sudhakaran Raja^2^, Thava Palanisami^1*^.

*1.* *Environmental Plastics Innovation Cluster, Global Innovative Centre for Advanced Nanomaterial, The University of Newcastle, Newcastle, NSW, Australia*

*2. Aquaculture Biotechnology Laboratory, School of Bio-Sciences and Technology, Vellore Institute of Technology, Vellore, India*

*3. School of Biomedical Sciences and Pharmacy, The University of Newcastle/ Priority Research Centre for Healthy Lungs, Hunter Medical Research Institute, The University of Newcastle, Newcastle, NSW, Australia*

*4. Department of Environmental Sciences, University of Kerala, Kerala, India*

***Corresponding Author**: Dr. Thava Palanisami, Environmental Plastics Innovation Cluster (EPIC), Global Innovative Centre for Advanced Nanomaterials (GICAN), The University of Newcastle, Callaghan, NSW, 2308, Australia.

Email: [thava.newcastle@gmail.com](mailto:thava.newcastle@gmail.com)

**Figure S1**: Isolated microplastics from the salt samples (Both fluorescent and normal images (40x magnification))


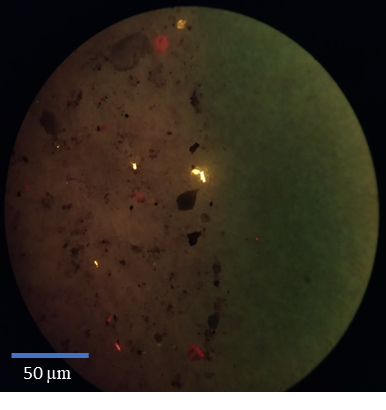

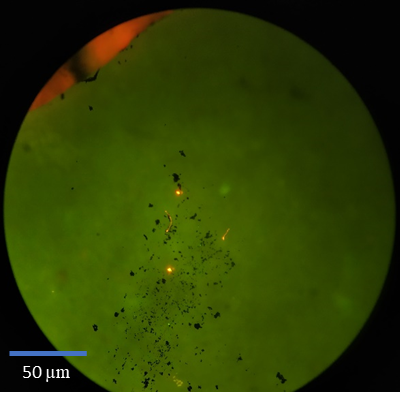


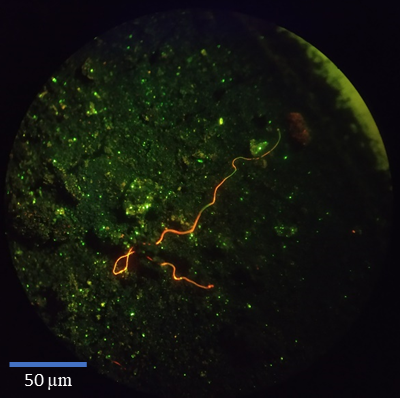


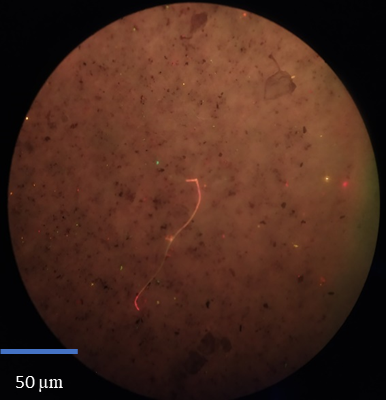

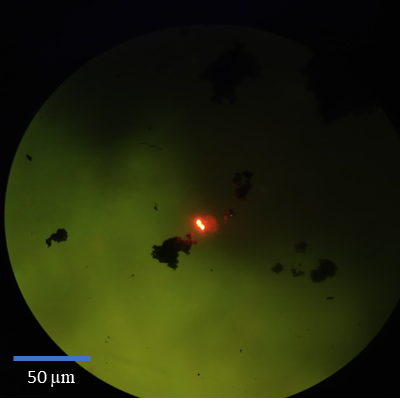


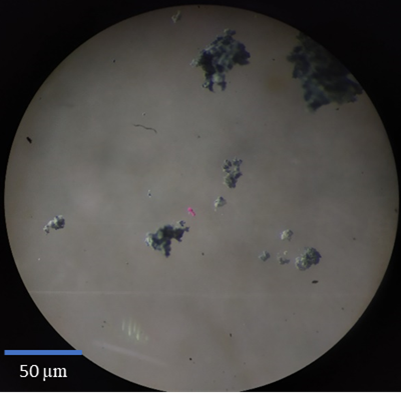


50 µm


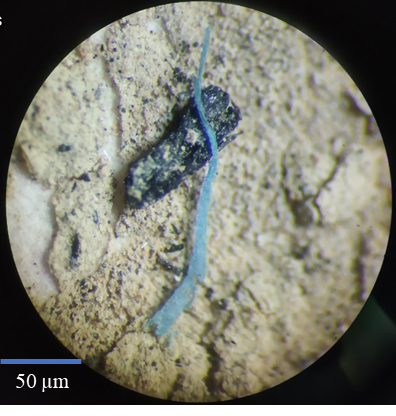

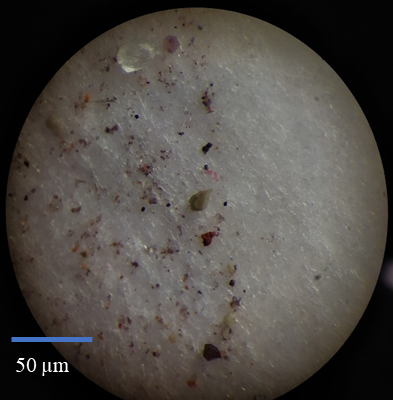

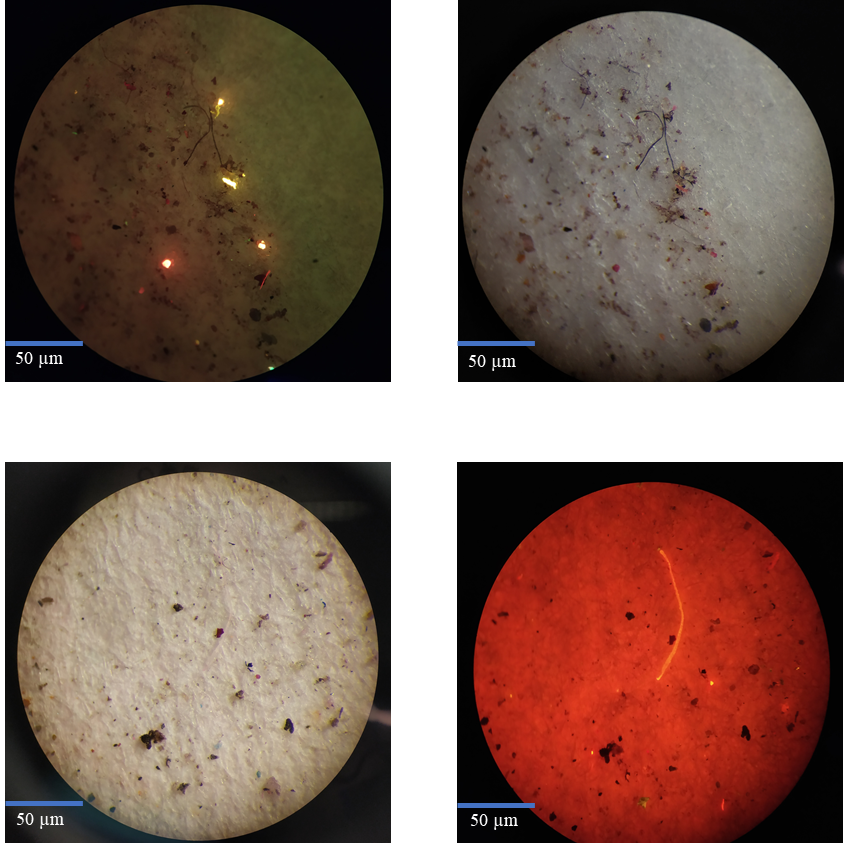


50 µm

**Figure S2:** EDS data of representative samples

1. **Particle 1**


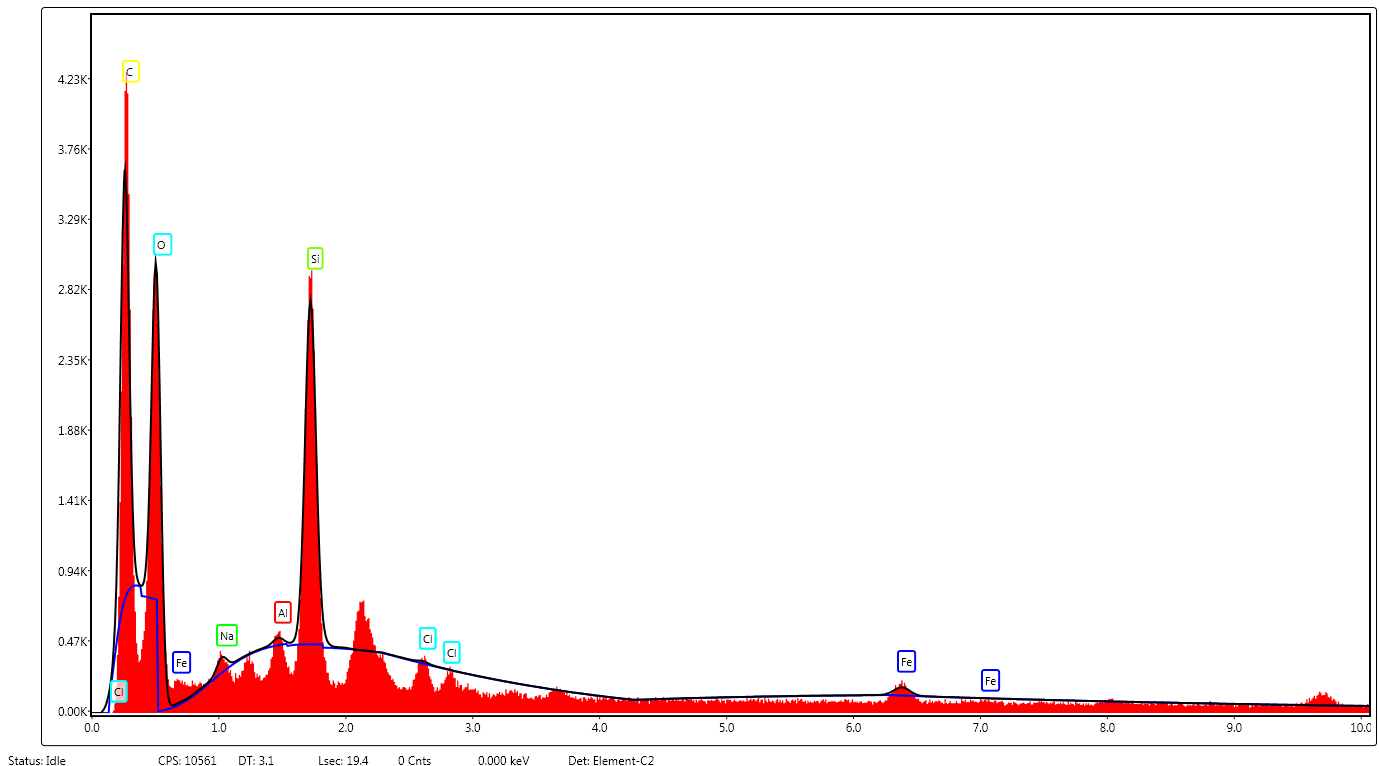


| **Element** | **Weight %** | **Atomic %** | **Error %** |
| --- | --- | --- | --- |
| C | 46.81 | 56.06 | 8.35 |
| O | 43.83 | 39.4 | 9.82 |
| Si | 8.29 | 4.24 | 3.94 |
| Cl | 0.07 | 0.03 | 59.85 |
| Fe | 1 | 0.26 | 21.37 |

1. **Particle 2**


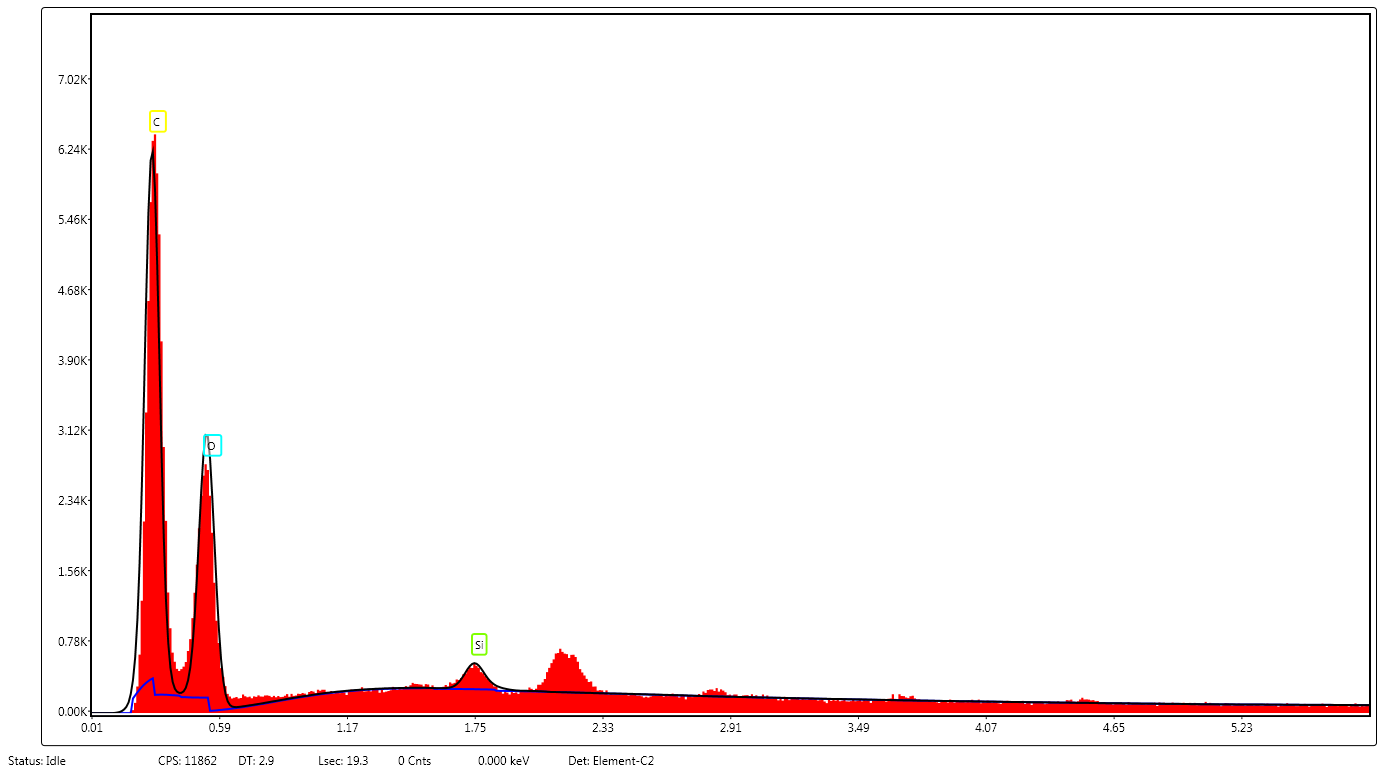


| **Element** | **Weight %** | **Atomic %** | **Error %** |
| --- | --- | --- | --- |
| C | 52.85 | 60.12 | 5.87 |
| O | 46.11 | 39.37 | 9.87 |
| Si | 1.05 | 0.51 | 9.19 |

1. **Particle 3**


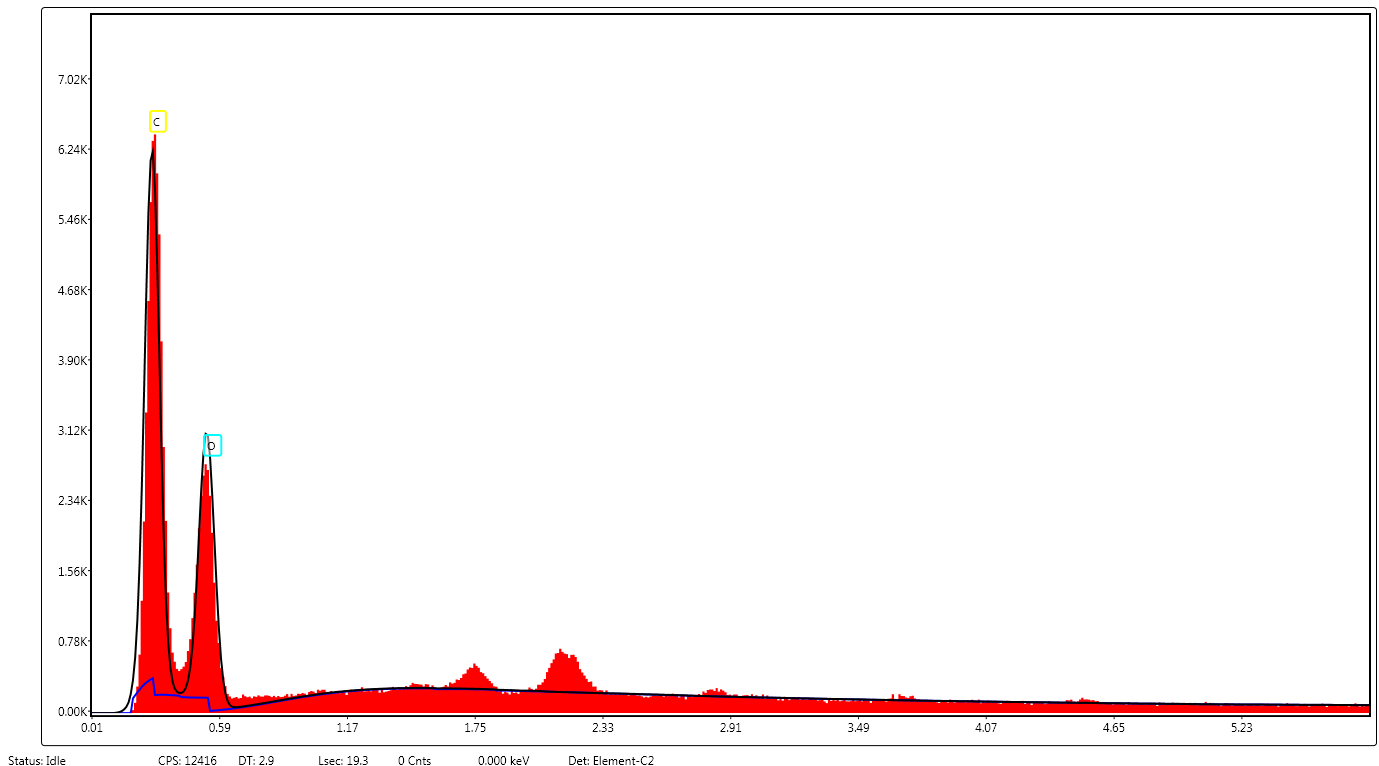


| **Element** | **Weight %** | **Atomic %** | **Error %** |
| --- | --- | --- | --- |
| C | 52.09 | 59.16 | 5.48 |
| O | 47.91 | 40.84 | 9.82 |

**Figure S3**: Characterisation and identification of isolated microplastics using Bruker Polymer ATR-FTIR Polymer Library

1. **Acrylonitrile butadiene styrene**

1. **Polypropylene**


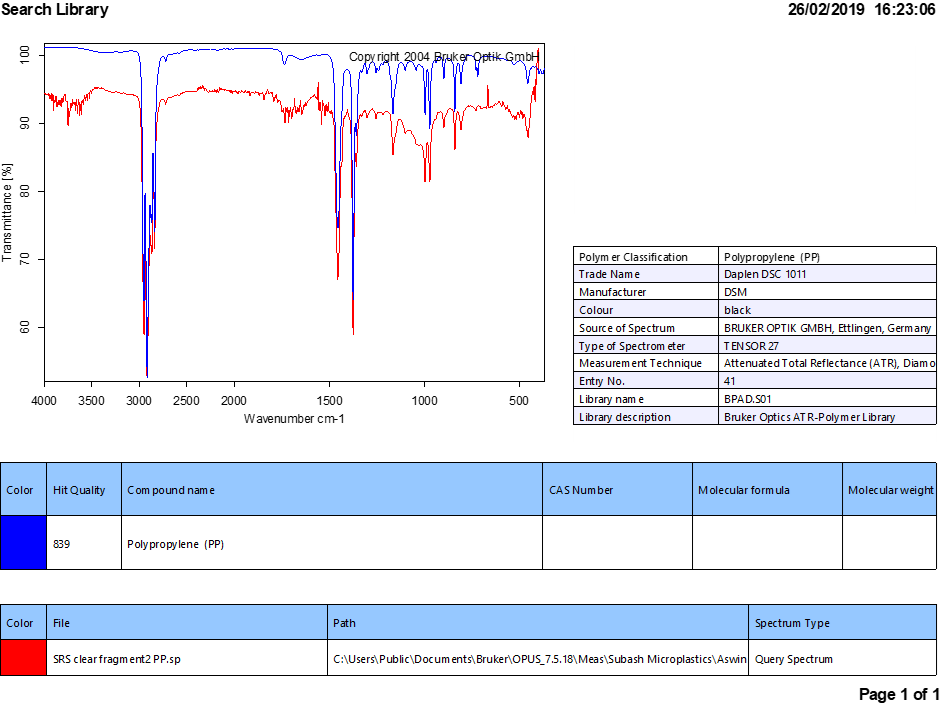


1. **Polyamide**

1. **Polyurethane**

1. **Cellulose acetate**

1. **Polyester**

1. **Polyethylene Terephthalate**

1. **Polyvinyl chloride**

1. **Polyethylene**

1. **Rayon**

**Figure S4:** Chemical composition of isolated microplastics in salt samples on different continents (based on 11 studies): A Review

The Pie chart represents the abundance of isolated polymer from salt across different continent


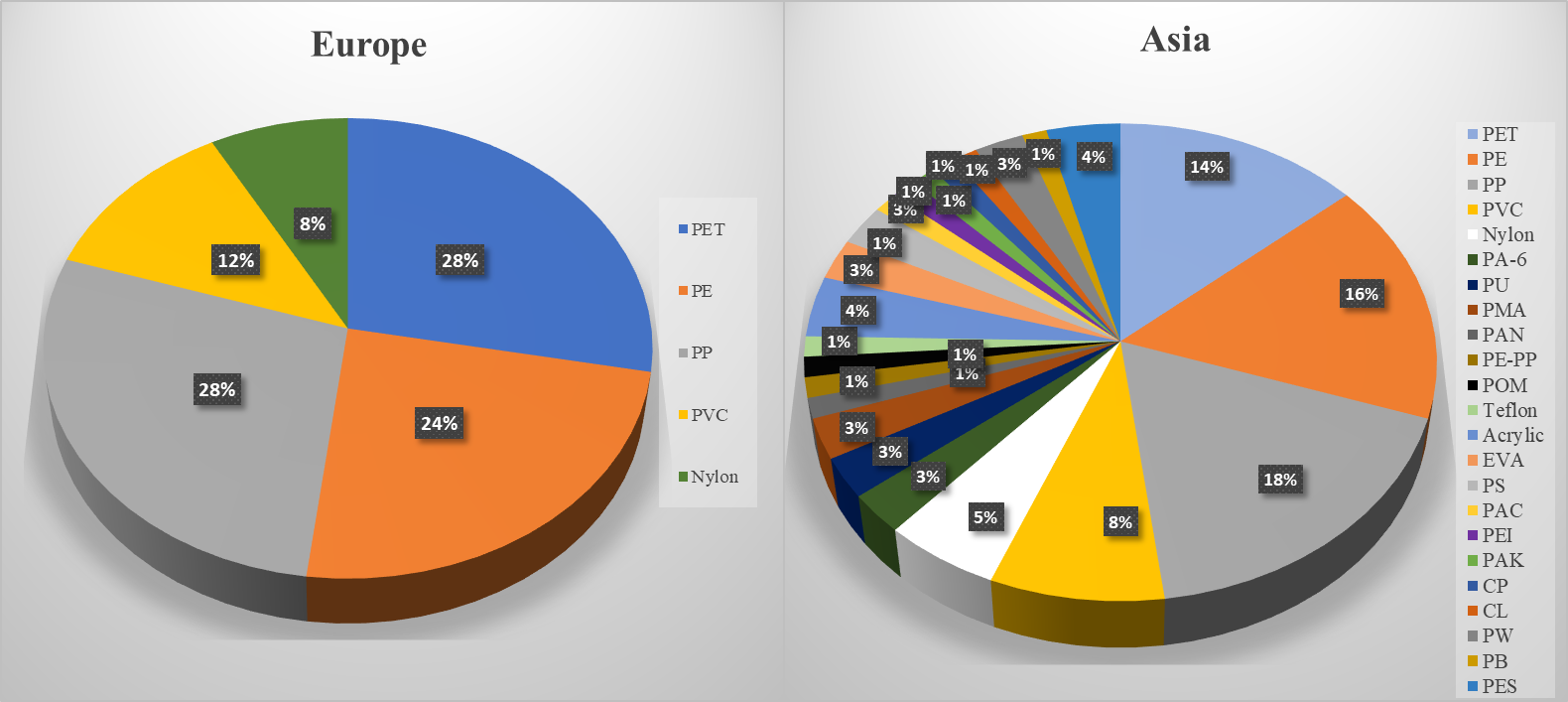


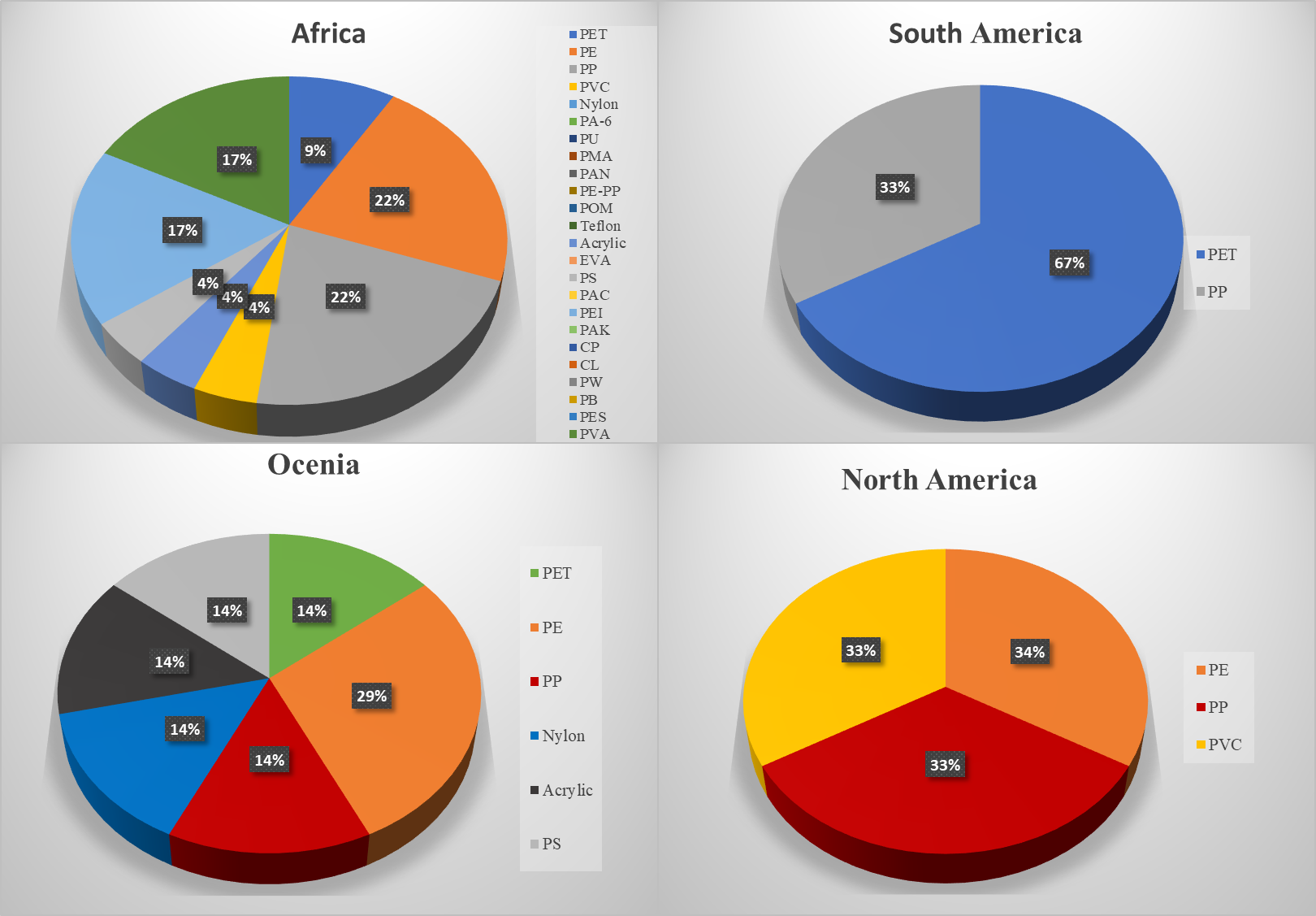


**Abbreviation**: Polyacetal(PAC), polyetherimide(PEI),ethylene vinyl acetate(EVA), cellulose(CL), polymethylacrylate (PMA), Polyethylene terephthalate (PET), polyethylene (PE), polypropylene (PP), cellophane (CP),Polystyrene (PS), polyester (PES),polyamide-6 (PA-6), paraffin wax (PW), phenoxy resin (PR), polyacrylate (PA), polycarbonate (PC), polyurethane (PU), polyvinylchloride (PVC), polyacrylonitrile (PAN), poly(1-butene) (PB), polymethyl methacrylate(PMMA), polyalkene (PAK),PE and PP copolymer,

**Figure S5: Chemical composition of isolated microplastics in salt samples vs country-based studies (based on 11 studies)**

**Figure S6**: Microplastics found in salt across the globe

**
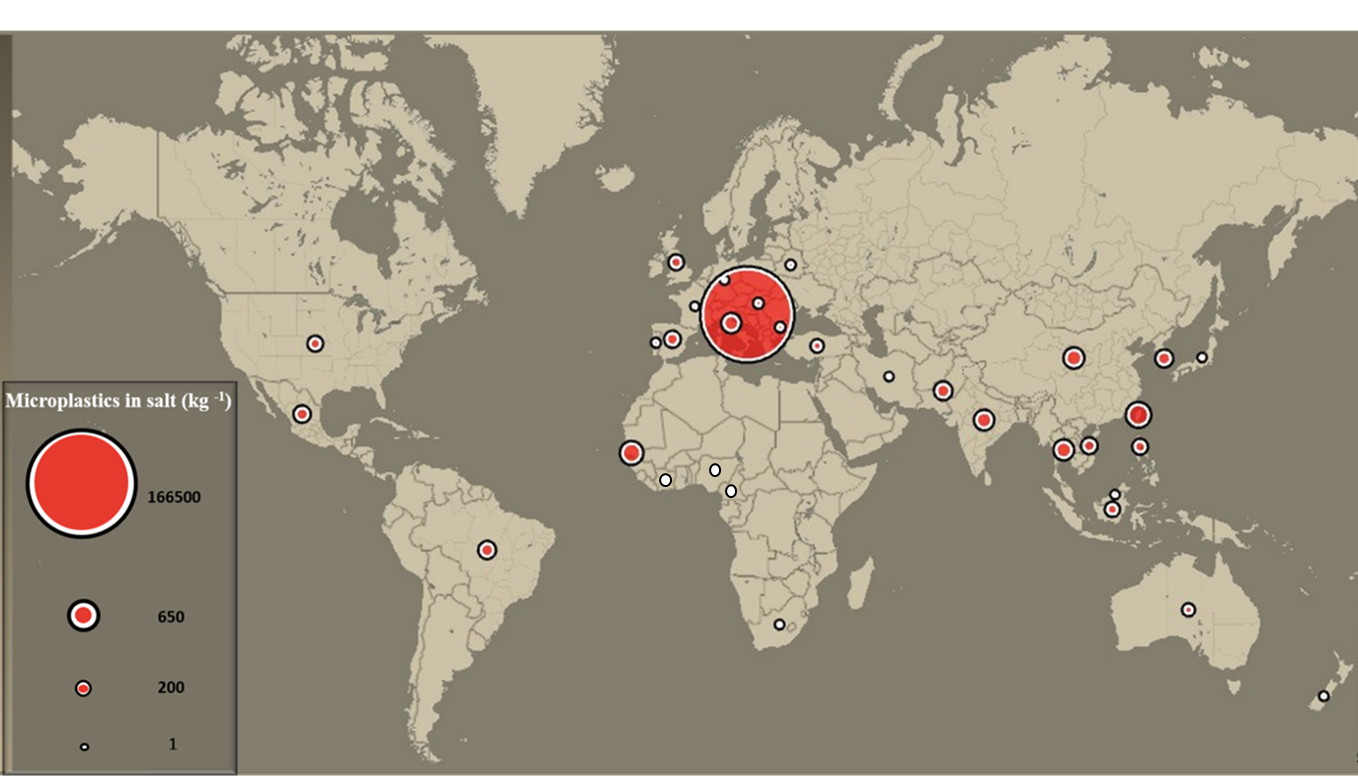
**

**Quantification of isolated microplastics from salt samples across the globe. The figure represents the average of different studies (Fadare, Okoffo, & Olasehinde, 2021; Gündoğdu, 2018; Iñiguez, Conesa, & Fullana, 2017; Karami et al., 2017; Kim, Lee, Kim, & Kim, 2018; Kosuth, Mason, & Wattenberg, 2018; Lee, Kunz, Shim, & Walther, 2019; Renzi & Blašković, 2018; Sathish, Jeyasanta, & Patterson, 2020; Seth & Shriwastav, 2018; Yang et al., 2015) in each country.**

**References**

Fadare, O. O., Okoffo, E. D., & Olasehinde, E. F. (2021). Microparticles and microplastics contamination in African table salts. *Marine Pollution Bulletin, 164*, 112006. doi:<https://doi.org/10.1016/j.marpolbul.2021.112006>

Gündoğdu, S. (2018). Contamination of table salts from Turkey with microplastics. *Food Additives & Contaminants: Part A, 35*(5), 1006-1014. doi:10.1080/19440049.2018.1447694

Iñiguez, M. E., Conesa, J. A., & Fullana, A. (2017). Microplastics in Spanish table salt. *Scientific Reports, 7*(1), 1-7.

Karami, A., Golieskardi, A., Choo, C. K., Larat, V., Galloway, T. S., & Salamatinia, B. (2017). The presence of microplastics in commercial salts from different countries. *Scientific Reports, 7*, 46173.

Kim, J.-S., Lee, H.-J., Kim, S.-K., & Kim, H.-J. (2018). Global Pattern of Microplastics (MPs) in Commercial Food-Grade Salts: Sea Salt as an Indicator of Seawater MP Pollution. *Environmental Science & Technology, 52*(21), 12819-12828.

Kosuth, M., Mason, S. A., & Wattenberg, E. V. (2018). Anthropogenic contamination of tap water, beer, and sea salt. *PLoS One, 13*(4), e0194970.

Lee, H., Kunz, A., Shim, W. J., & Walther, B. A. (2019). Microplastic contamination of table salts from Taiwan, including a global review. *Scientific Reports, 9*(1), 10145. doi:10.1038/s41598-019-46417-z

Renzi, M., & Blašković, A. (2018). Litter & microplastics features in table salts from marine origin: Italian versus Croatian brands. *Marine Pollution Bulletin, 135*, 62-68. doi:<https://doi.org/10.1016/j.marpolbul.2018.06.065>

Sathish, M. N., Jeyasanta, I., & Patterson, J. (2020). Microplastics in Salt of Tuticorin, Southeast Coast of India. *Archives of environmental contamination and toxicology, 79*(1), 111-121. doi:10.1007/s00244-020-00731-0

Seth, C. K., & Shriwastav, A. (2018). Contamination of Indian sea salts with microplastics and a potential prevention strategy. *Environmental Science and Pollution Research, 25*(30), 30122-30131.

Yang, D., Shi, H., Li, L., Li, J., Jabeen, K., & Kolandhasamy, P. (2015). Microplastic Pollution in Table Salts from China. *Environ Sci Technol, 49*(22), 13622-13627. doi:10.1021/acs.est.5b03163
